# Identification and Evolution of the WUSCHEL-Related Homeobox Protein Family in Bambusoideae

**DOI:** 10.3390/biom10050739

**Published:** 2020-05-09

**Authors:** Xiangyu Li, Juan Li, Miaomiao Cai, Huifang Zheng, Zhanchao Cheng, Jian Gao

**Affiliations:** International Center for Bamboo and Rattan, Key Laboratory of Bamboo and Rattan Science and Technology, State Forestry and Grassland Administration, Beijing 100102, China; lxy@icbr.ac.cn (X.L.); lijuan@icbr.ac.cn (J.L.); cmm@icbr.ac.cn (M.C.); zhenghuifang@icbr.ac.cn (H.Z.); czc@icbr.ac.cn (Z.C.)

**Keywords:** bambusoideae, *WOXs*, polyploidization, selection pressure

## Abstract

Bamboos (Bambusoideae) are fast-growing species due to their rapid growth rate and ability to reproduce annually via cloned buds produced on the rhizome. WUSCHEL-related homeobox (WOX) genes have been reported to regulate shoot apical meristem organization, lateral organ formation, cambium and vascular proliferation, and so on, but have rarely been studied in bamboos. In this study, the *WOXs* of both herbaceous bamboo species (12 *OlaWOXs* and nine *RguWOXs*) and woody bamboo species (18 *GanWOXs*, 27 *PheWOXs*, and 26 *BamWOXs*) were identified and categorized into three clades based on their phylogenetic relationship—ancient, intermediate, or WUS clade. Polyploidy is the major driver of the expansion of the bamboo *WOX* family. Eight conserved domains, besides the homeodomain, were identified by comparatively analyzing the WOXs of dicot and monocot species. Intensive purifying selection pressure in the coding region of specific domains explained the functional similarity of *WOXs* between different species. For Bambusoideae *WOXs*, polyploidy is the major driver of the expansion of the *WOX* family. Stronger purifying selection was found in orthologous *WOXs* of Bambusoideae, especially for *WOX4s* and *WOX5s*, which are conserved not only at the translational levels, but also at the genome level. Several conserved *cis*-acting elements were discovered at similar position in the promoters of the orthologous *WOX*s. For example, AP2/ERF protein-binding elements and B3 protein-binding elements were found in the promoters of the bamboo *WOX4*, while MYB protein-binding elements and Dof protein-binding elements were found in the promoters of bamboo WOX5, and MADS protein-binding sites was found in the promoters of bamboo *WUS*, *WOX3,* and *WOX9*. These conserved positions may play an important role in regulating the expression of bamboo *WOXs*. Our work provides insight into the origin and evolution of bamboo *WOXs*, and will facilitate functional investigations of the clonal propagation of bamboos.

## 1. Introduction

Bamboos are among the fastest-growing plants on Earth, and the nearly 1500 described bamboo species are native to all continents except Antarctica and Europe [1,2]. As a potential lignocellulosic material, due to its availability, good material properties, and high yield resources, bamboo is as an important pillar of the forest industry for many Asian countries, as well as some in Africa and Latin America. Bamboo contributes great economic and ecological benefits to the world as a fast-growing wood, as well as a source of food and an industrial material. Bamboo rarely flowers, with flowering cycles of up to 120 years [3]. Bamboo forests mainly spread by clonal reproduction, relying on the meristem of the nutritive organ to create shoots of new individuals. Taking the study of the most economically and ecologically rewarding bamboo, moso bamboo (*Phyllostachys edulis*), as an example, underground growth includes the extension of the rhizome, the development of the rhizome root, and the development of lateral buds located in the internode of the rhizome. The development of lateral buds is extraordinarily important for bamboo development, which proceeds from the dormant buds to the active buds, and then to new shoot development [4]. It takes a long time, even years, for dormant bud development to provide active shoots underground. However, the aboveground growth, from shoots breaking through the soil to young bamboo, finishes in two months, with a high growth rate that is as fast as 1 m per day [5], which illustrates how rapidly bamboo forests can spread.

Bamboos fall into four monophyletic lineages with different ploidy levels according to the number of basic sets of bamboo chromosomes, diploid herbaceous bamboos (2n = 20–24), temperate tetraploid woody bamboos (2n = 46–48), neotropical tetraploid woody bamboos (2n = 40–48), and palaeotropical hexaploid woody bamboos (2n = 70–72) (reviewed in [1]). Recently, Guo et al. (2019) reported the draft genomes of two diploid herbaceous bamboo species (*Olyra latifolia,* 2n = 2x = 22; *Raddia guianensis,* 2n = 2x = 22), one neotropical tetraploid woody bamboo (*Guadua angustifolia,* 2n = 4x = 46), and one palaeotropical hexaploid woody bamboo (*Bonia amplexicaulis,* 2n = 6x = 72). A distinct subgenome was identified in these polyploid and *Phyllostachys edulis* (temperate tetraploid woody bamboo, 2n = 4x = 48). The polyploidizations of woody bamboos gave rise to four distinctly ancestral subgenomes, namely, A, B, C, and D, resulting in the allohexaploid palaeotropical *Bonia amplexicaulis* (AABBCC), the allotetraploid neotropical *Guadua angustifolia* (BBCC), and the allotetraploid temperate *Phyllostachys edulis* (CCDD) [1]. This significant research provides the molecular evidence for the insights into the origin of woody bamboos at the subgenome level, and the basis to study gene family evolution during allopolyploidization.

The WUSCHEL-related homeobox (WOX) genes belong to the homeobox transcription factor (HB TF) family, which encode typical plant transcription factors sharing a DNA-binding domain with a conserved stretch of 60-66 residues, the Helix-loop-helix-turn-helix structure [6,7,8]. The other regions of the WOX proteins are highly divergent [9], on account of which WOX proteins can be divided into three separate clades, namely the ancient clade, the intermediate clade, and the WUS clade [8]. In *Arabidopsis*, the WOX family consists of 15 members, namely, *WUSCHEL* (*WUS*) and *WOX1*-*WOX14* [10]. In *Arabidopsis*, the ancient clade harbors WOX10, 13, and 14 proteins, the WUS clade harbors WUS and WOX 1-7 proteins, and the intermediate clade harbors WOX8, 9, 11, and 12 proteins.

In vascular plants, *WOXs* play important roles in various processes, including maintenance of the root and shoot apical meristems, vascular development, embryogenesis development, and adventive organ development [10,11,12,13]. *WUS*, *WOX4*, and *WOX5* regulate stem cell maintenance in the shoot and floral meristems, cambium, and root meristem [11,14,15,16,17,18], and *WOX3* contributes to marginal and plate meristem activity of leaves [19,20]. *WOX8* (*STIMPY-LIKE*, *STPL*) and *WOX9* (*STIMPY*, *STIP*) of *Arabidopsis thaliana* are essential for embryo patterning and vegetative SAM maintenance [10,21,22]. *WOX2* in *Arabidopsis thaliana* promotes cotyledon boundary formation during early embryogenesis, redundantly with *WOX8* [22]. *WOX11/12* activate the expression of *WOX5* for root primordia initiation during de novo organogenesis [23,24]. In *Arabidopsis*, *WUSCHEL* (*WUS*) maintains the stem cell niche in the shoot apical meristem (SAM) by partnering with the WUS-CLAVATA3 (WUS-CLV3) signaling pathway [12,14,25,26]. However, the rice ortholog of *WUS*, *TILLERS ABSENT1* (*TAB1*), is not expressed in the SAM and does not participate in stem cell proliferation, but is transiently expressed in the early stages of axillary meristem development and plays a crucial role by interacting with the *CLAVATA3* orthologous gene, *FLORAL ORGAN NUMBER2* (*FON2*), in rice axillary meristem development [13,17]. *WOX4* of rice acts as a positive regulator of stem cell maintenance in the SAM and axillary meristem [18]. Taken together, the WOX family is essential for the establishment of plant architecture in various fundamental aspects, including the formation and maintenance of shoot and root apical meristems, the development of lateral organs, the maintenance and differentiation of cambium, floral formation, and the formation of polarity in the embryo.

*WOX* genes may play key roles during bamboo rhizome bud formation, as well as in the activation of dormant buds, the maintenance of shoot apical meristems, and the formation of adventitious roots during bamboo clonal reproduction, while the regulatory mechanisms underlying when and how meristems are established, maintained, and lost during bamboo regeneration are poorly understood. In the present study, we identified 91 putative bamboo *WOXs* of herbaceous and woody bamboos. Phylogenetic analysis classified these *WOXs* into three clades. Specific motifs were identified by a comparative analysis of the proteins of *Phyllostachys edulis* (*P. edulis)* with *Arabidopsis thaliana*, *Populus trichocarpa*, *Oryza sativa*, *Zea mays*, and *Brachypodium distachyon*. The conserved specific domain of each clade was characterized and analyzed conjointly with phylogenetic analysis. Comparative analysis of *PheWOXs* with *WOXs* of two herbaceous bamboo species and three woody bamboo species was performed. Our study will serve as a foundation for future research on the functional roles of *WOXs* in Bambusoideae, as well as provide insight into the essential roles of WOXs in *P. edulis* development.

## 2. Methods

### 2.1. Identification of the WOX Gene Family in Bambusoideae Genomes

WOXs of *Arabidopsis thaliana*, *Populus trichocarpa*, *Oryza sativa*, *Zea mays*, *Brachypodium distachyon*, *Micromonas pusilla*, *Micromonas sp.*, *Ostreococcus tauri*, and *Bathycoccus prasinos* were collected from PlantTFDB v4.0 (http://planttfdb.cbi.pku.edu.cn/family.php?fam=WOX) [27] and rechecked by NR (non-redundant protein sequence database) annotation and conserved homeodomain (HD) identification. WOXs from these species were used as queries to blast the *P. edulis* polypeptide dataset (ftp://parrot.genomics.cn/gigadb/pub/10.5524/100001_101000/100498/), the Olyra latifolia (*O. latifolia*) polypeptide dataset, the Raddia guianensis (*R. guianensis*) polypeptide dataset, the *Guadua angustifolia* (*G. angustifolia*) polypeptide dataset, and the *Bonia amplexicaulis* (*B. amplexicaulis*) polypeptide dataset (http://www.genobank.org/bamboo) with the threshold e-value of 10^−5^ [1,28]. The conserved domain database of NCBI was used to confirm the HD of putative WOX proteins [29]. Furthermore, the genome sequence of putative WOXs was obtained according to Gene ID of the genome information by TBtools [30].

### 2.2. Phylogenetic Tree Construction and Conserved Motif Analysis of WOXs

The multiple alignment of WOX proteins was performed using CLUSTAL_X software [31]. After manually removing the poorly aligned sequences and divergent regions of the WOX protein alignment, MEGA5 was used to construct a neighbor-joining tree of WOXs with a bootstrap assessment of 1000 replicates [32]. A maximum-likelihood tree was also constructed to validate the topologies. Multiple Em for Motif Elicitation (MEME Version 5.0.4, http://meme-suite.org/tools/meme) was used to identify conserved motifs among WOXs using the following parameters: the site distribution of zero or one site per sequence; motifs with a maximum number of 11; and motifs with a width of around 10–70 characters. The visualization of the tree and motif distribution was implemented by Evolview v2.0 [33].

### 2.3. Selective Pressure Analysis of Orthologous and Paralogous Genes

The non-synonymous substitution rate (Ka), the synonymous substitution rate (Ks), and the Ka/Ks of gene pairs were calculated using PAML [34]. To identify signals of positive selection of certain regions, a sliding window analysis of the Ka/Ks ratios was carried out with a window size of 60 Angstrom using SWAKK [35]. The divergence time of the homologous *WOXs* was calculated using the formula: T = Ks/2λ [36], with the divergence rate λ = 6.5 × 10^−9^ [2].

### 2.4. cis-Acting Element Analysis of the P. edulis WOX Promoter

The *WOX* promoter sequence 1500 bp upstream from the start codon (ATG) was extracted using TBtools [30]. The *cis*-acting element analysis was conducted based on the *cis-*acting element dataset of Plant Transcriptional Regulatory Map (PlantRegMap), which was derived from high-throughput assays of ChIP-seq and genome-wide TF footprinting [27,37]. The regulatory element distribution of the *WOX* promoter was analyzed with the threshold *p*-value of 10^−5^.

## 3. Results

### 3.1. Identification of the WOXs of Bambusoideae

Twenty-seven putative *WOX* genes of *Phyllostachys edulis* (*PheWOXs*) were identified in the *P. edulis* genome (Version 2) (Supplementary Table S1) [28]. Of these, 21 contain complete HDs with around 60 amino acid residues, and the other six code proteins with incomplete HDs at the N-terminal region which may impede DNA-binding, but show high similarity with WOX [6,8]. In addition, the *WOX* genes of four other bamboos (*O. latifolia*, *R. guianensis*, *G. angustifolia*, and *B. amplexicaulis*) were also identified from draft genomes [1], namely 12 *WOXs* of *O. latifolia* (*OlaWOXs*), nine *WOXs* of *R. guianensis* (*RguWOXs*), 18 *WOXs* of *G. angustifolia* (*GanWOXs*), and 26 *WOXs* of *B. amplexicaulis* (*BamWOXs*) (Supplementary Table S1). The number of ortholog of *WOX* is weakly correlated with Bambusoideae ploidy (Table 1). Polyploidy is a major driver of plant evolution [38], and drives family expansion and functional divergence of *WOXs*. The number of WOX orthologs generally increases with ploidy level as would be expected with increase genome copies. The single exception is hexaploid *B amplexiaulis* with 26 WOX orthologs compared to 27 orthologs in the tetraploid genome of *P. edulis*. This lower than expected number is likely due to the lower sequence quality and assembly of *B. amplexicaulis* (848 Mb of scaffold with an N50 length of 1.76 Mb) compared to *P. edulis* (1886 Mb of scaffold with an N50 length of 79.90 Mb) [1,28].

The WOX proteins of *Arabidopsis thaliana*, *Populus trichocarpa*, *Oryza sativa*, *Zea mays*, and *Brachypodium distachyon* were also collected from PlantTFDB v4.0 and manually examined (Supplementary Table S2). After removing the redundant sequences with the same chromosome locations, 15 *WOXs* of *Arabidopsis thaliana* (*AtWOXs*), 18 *WOXs* of *Populus trichocarpa* (*PotriWOXs*), 13 WOXs of *Oryza sativa* (*OsWOXs*), 20 WOXs of *Zea mays* (*ZmWOXs*), and 13 WOXs of *Brachypodium distachyon* (*BradiWOXs*) were used for later analysis. All of the candidate WOX proteins contained a domain matching IPR001356 (homeobox domain, HD) of the Interpro database. The number of *WOXs* varied in different species (Table 1).

The nomenclature of the *WOXs* genes varies among different species, resulting in confusion regarding the understanding of gene function. For example, *Os03g0325600* of rice was named both *WOX6* and *WOX12B* (https://rapdb.dna.affrc.go.jp/viewer/gbrowse_details/irgsp1?name=Os03g0325600). In this study, the *WOXs* were named after their orthologs of *AtWOXs* based on the conserved domain of the different clades and their evolutionary relationships. The notations a, b, and c were appended to differentiate highly similar paralogs in the same species (Supplementary Table S1).

### 3.2. Identification of Conserved Domains of the WOX Family

**Figure 1:**
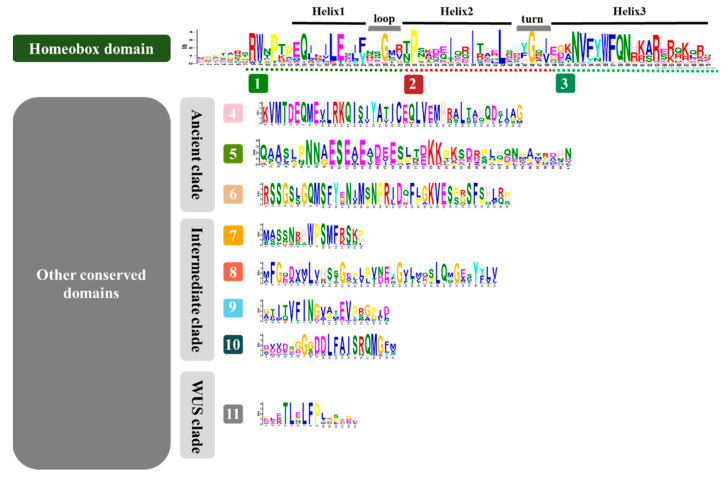
The conserved domains identified in the WUSCHEL-related homeobox (WOX) family. Eleven conserved domains were identified among 15 AtWOXs, 18 PotriWOXs, 13 OsWOXs, 20 ZmWOXs, 27 PheWOXs, 13 BradiWOXs, 12 OlaWOXs, 9 RguWOXs, 18 GanWOXs, and 26 BamWOXs by Multiple Em for Motif Elicitation (MEME). Motifs 4-6 were identified in the WOXs belonging to the ancient clade. Motifs 7-10 were identified in the WOXs belonging to the intermediate clade. Motif 11 was found in the WOX belonging to the WUS clade.

**Figure 2:**
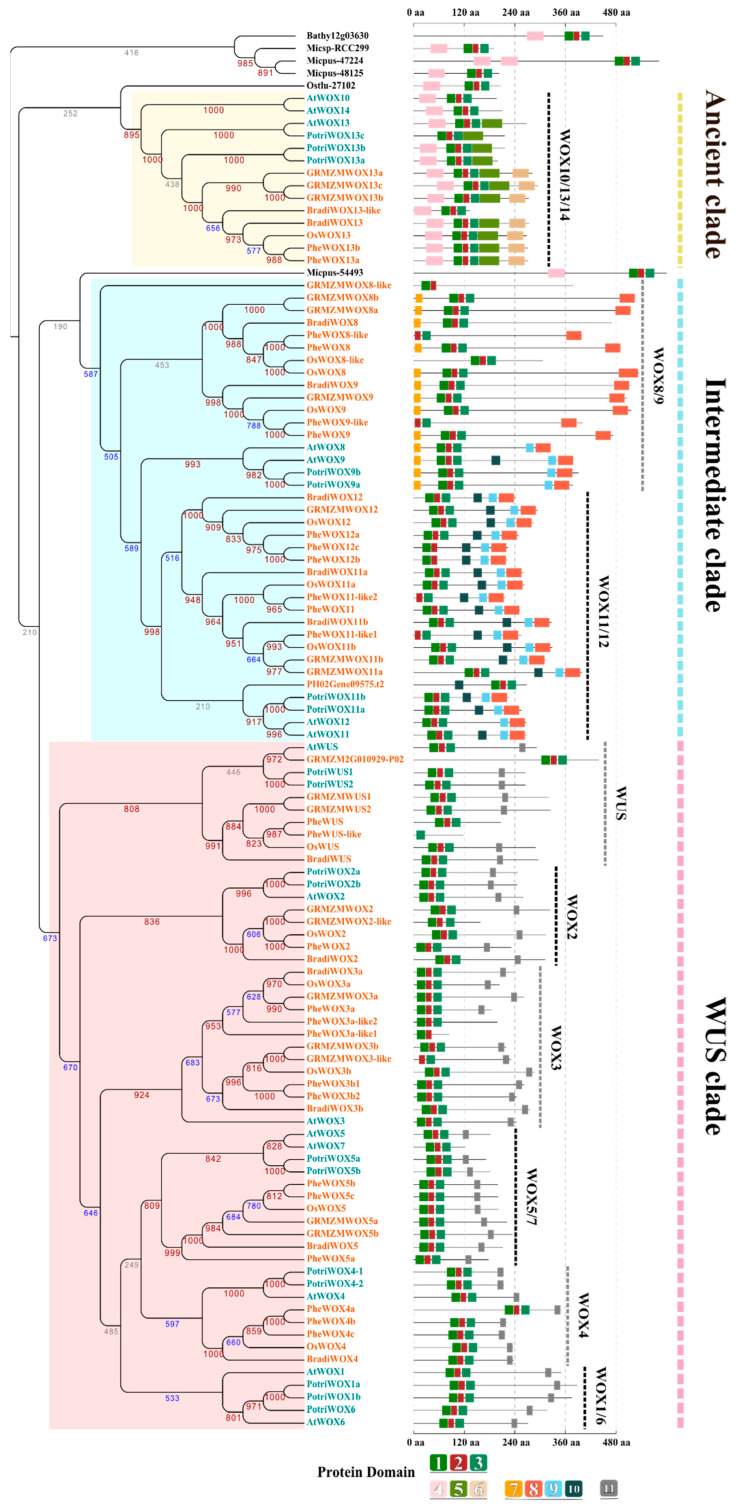
The phylogenetic tree of WOXs from dicots and monocots. The neighbor-joining tree was constructed using amino acid sequences of WOXs from *Arabidopsis thaliana* (At), *Populus trichocarpa* (Potri), *Oryza sativa* (Os), *Zea mays* (Zm), *Brachypodium distachyon* (Bradi), *Micromonas pusilla* (Micpu), *Micromonas sp.* (Micsp), *Ostreococcus tauri,* (Osta), and *Bathycoccus prasinos* (Bathy), with bootstrap assessment of 1000 replicates. Each clade was assigned a different colored background. Leaf colors of black, orange, and cyan stand for WOXs of chlorophytes, monocots, and dicots, respectively. The graphic symbols of the conserved motifs are colored and termed the same as in Figure 1.

To better understand the protein features of WOXs, the conserved motifs of these WOXs were searched by MEME. Eleven conserved domains were found among the 171 WOX proteins (Figure 1). Motifs 1–3 are residues of HDs that contain three helices spaced by one loop and one turn [6]. Conserved amino acids of HDs [6,9,39], Q, L, and E of helix 1, P, I, and L of helix 2, N, V, W, F, Q, N, and R of helix 3, and G of the turn were identified (Figure 1). In addition to HDs, eight specific domains were found across the WOXs of dicots and monocots, including Motifs 4–6 found in the ancient clade, Motifs 7–10 found in the intermediate clade, and Motif 11 found in the WUS clade (Figure 1). Most of the members of each clade share one or more conserved domains, in addition to HDs (Figure 2). These domains were conserved during evolution of the family and may play a particular role in protein function.

### 3.3. The Evolution of the WOX Family

To further understand the evolution of *WOX* genes, phylogenetic analysis of 103 WOXs (*AtWOXs*, *PotriWOXs*, *OsWOXs*, *ZmWOXs*, *BradiWOXs*, and *PheWOXs*) was carried out (Figure 2). The polypeptides used to construct the phylogenetic tree are shown in Supplementary Dataset S1. The maximum likelihood tree of the *WOX* family showed topologies similar to the neighbor-joining tree (Supplementary Figure S1). The WOX13 subfamily was considered an outgroup [40,41]. These WOXs divided into three distinct clades, as described before, namely, the ancient clade, intermediate clade, and WUS clade [8]. Each clade was well-supported by high bootstrap values, except for the ancient clade, which was consistent with previous studies [9,42,43]. To further investigate the evolutionary relationship of the WOX family, nine sub-groups were identified based on their phylogenetic topology, namely, WOX10/13/14 of the ancient clade, WOX8/9 and WOX11/12 of the intermediate clade, and WUS, WOX2, WOX3, WOX5/7, WOX4, and WOX1/6 of the WUS clade (Figure 2). This is consistent with previous studies [42].

*WOXs* underwent gene expansion in both dicots and monocots, particularly for the WUS and intermediate clades. The WUS and intermediate clades contained more genes than the ancient clade (Table 1). The evolutionary topologies of the sub-groups were clearly separated between dicots and monocots, and the topologies of WOX10/13/14 between dicots and monocots were hierarchical. Members of WOX11/12, WUS, WOX2, WOX3, WOX5/7, and WOX4 formed two separate branches of monocots and dicots with higher support values. This suggests that the genes of WOX10/13/14 occurred before the divergence of monocots and dicots and varied only slightly as the categories differentiated. The ancient members of WOX11/12, WUS, WOX2, WOX3, WOX5/7, and WOX4 also existed before the divergence of monocots and dicots, and diversified with different evolutionary rates after their divergence [44].

### 3.4. Specific Domain Evolution of WOXs from Different Plants

The functional domains of transcription factors always appear to diverge at reduced rates because of their critical role in protein function [45]. The HD is the basic feature of HB TFs and originated before the divergence of eukaryotes [7,8]. Some conserved amino acid sites of the HD were identified among plants (Figure 2), which might limit the variation of WOXs. Although the HD is conserved, the position of the WOX proteins of Chlorophyta and higher plants is different. In Chlorophyta, the HD is located closer to the C-terminal of proteins, while the HD of higher plant is far from the C-terminal due to the new conserved domains formed in the C-terminal (Figure 2). The HDs of GRMZM2G010929-P02 and PH02Gene09575.t2 are also located near the C-terminal (Figure 2). These two genes were clustered in WOX11/12 and WUS with low support.

Besides conserved HDs, other conserved domains of WOXs formed during the expansion of the WOX family. WOXs of each clade contain one or more specific domains (Figure 2). Although genes of the ancient clade divided into sub-branches close to Chlorophyta, the WOXs of dicots and monocots were in highly segregated clusters in the ancient clade. Motif 4, around 40 amino acid residues long, was found among the WOX10/13/14 of dicots and monocots. The homologous genes of *AtWOX10* and *AtWOX14* were not identified in other species. The specific domains of AtWOX10 and AtWOX14 are similar to Micpus-48125 of *Micromonas pusilla*, Micsp-RCC299 of *Micromonas sp.,* and Ostlu-27102 of *Ostreococcus tauri*, which contain HDs and Motif 4 (Figure 2). Motif 5 was discovered in WOX13s, but not in WOX10/14s. Motif 6 was only found in the C-terminal of the WOX13s of *Oryza sativa*, *Zea mays*, *Brachypodium distachyon*, and bamboo, but not in *Populus trichocarpa* and *Arabidopsis thaliana* However, certain conserved amino acids of Motif 6 were found in the same position of AtWOX13 and PotriWOX13s (Supplementary Figure S2). We speculate that the origins of Motifs 5 and 6 are the same in dicots and monocots, but varied at different evolutionary rates.

The intermediate clade included WOX8/9s and WOX11/12s. Although Micpus-54493 clustered in the branch sister to intermediate clade, the bootstrap value was quite low (Figure 2). The topological structure of the intermediate clade divided into four sub-branches based on evolution, consisting of WOX8/9 of monocots, WOX8/9 of dicots, WOX11/12 of monocots, and WOX11/12 of dicots (Figure 2). Four motifs were found in the intermediate clade. Motif 7 was found at the N-terminal of most WOX8/9 proteins, which was not existent in the WOX11/12 proteins (Figure 2). Motifs 8 and 9 were located at the C-terminal of some WOX8/9s and all WOX11/12s. Moreover, AtWOX9 contained low-resolution Motif 10 in the same position as WOX11/12s (Figure 2). The loss/appearance of these domains might be associated with the functional differentiation of WOX8/9 and WOX11/12 in dicots and monocots.

The WUS clade included more genes than the other two clades. There were only two conserved motifs identified in the WUS clade, namely, the HD near the N-terminal and Motif 11 located at the C-terminal. Motif 11, containing the amino acid residue TLXLFP, is also called the WUS box and has been proven to be an important domain for repressor activity of the WUS clade proteins [46]. The WUS box has played an important role in the conserved function of the WUS clade. Indeed, the evolution of the WUS clade might be tied to the function of Motif 11. We could not find the WUS box in *PheWUS*, which might have a different function in *P. edulis* compared to other species.

### 3.5. Evolution of WOXs in Bambusoideae with Different Ploidies

**Figure 3:**
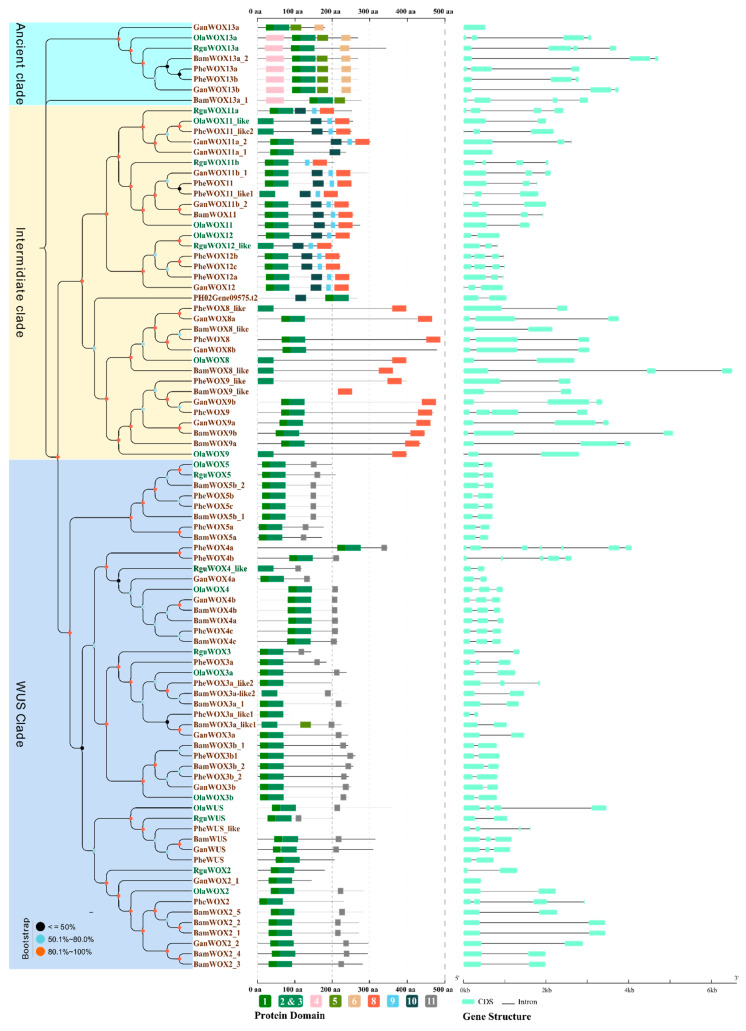
The phylogenetic tree of Bambusoideae *WOXs*. The neighbor-joining tree was constructed by the full-length CDS of Bambusoideae WOXs with bootstrap assessment of 1000 replicates. Each clade was assigned a different colored background. Green leaves of the phylogenetic tree stand for *WOXs* of herbaceous bamboos. Brown leaves of the phylogenetic tree stand for *WOXs* of woody bamboos. The protein domains and gene structures are drawn following each *WOX*. The name of each domain is the same as in Figure 1.

Bamboos have diverse polyploidies. The evolution of Bambusoideae is driven by polyploidization [1]. The absolute copy number of WOXs increased in polyploid bamboo species compared with diploid species. The unrooted phylogenetic tree was constructed using 91 Bambusoideae *WOX* coding sequence (CDS) with the assessment of bootstrap value. The phylogenetic tree showed that *WOXs* of Bambusoideae were also classified into the ancient clade, intermediate clade, and WUS clade, with a high support value of over 80%. Specific motif domains and gene structures were also identified (Figure 3). Three *WOX*-like genes (*BamWOX8like*, *BamWOX9like*, and *PheWUSlike*) of the 91 Bambusoideae were found to encode proteins with no HD (Figure 3), whereas 12 had partial HDs of motifs 2 and 3 at their N-terminal (Figure 3).

From the specific domain distribution observed, WOX13s of Bambusoideae were highly conserved, which was reflected not only in the protein length, but also in the domain position. However, *WOX13s* of Bambusoideae showed diverse numbers and lengths of exons and introns (Figure 3). Two groups—*WOX4s* and *WOX5s*—of Bambusoideae had high similarity at the protein level, and were also significantly more conserved at the genome level in different polyploidies (Supplementary Figures S3 and S4). These findings might be related to the important and conserved function of *WOX4s*, *WOX5s,* and *WOX13s* in Bambusoideae.

### 3.6. The Selective Constraints of Homologous Genes within and between Species

To investigate the evolutionary rate of WOX genes, the Ka/Ks ratios of sub-groups within and between species were calculated. A ratio of Ka/Ks > 1 indicates accelerated evolution with positive selection, a ratio =1 indicates neutral selection, and a ratio <1 indicates negative/purifying selection. This analysis showed that the *WOX* family had strong purifying selection pressure, as almost all of Ka/Ks ratios of the homologous *WOXs* detected were <1 (Supplementary Table S3).

**Figure 4:**
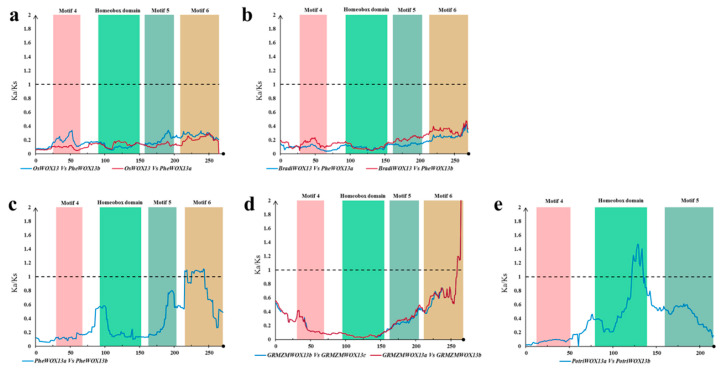
Sliding window of the *WOX13* genes of the ancient clade. (**a**) The sliding window of orthologous *WOX13s* between *P. edulis* and *Oryza sativa*. (**b**) The sliding window of orthologous *WOX13s* between *P. edulis* and *Brachypodium distachyon*. (**c**–**e**) The sliding window of paralogous *WOX13s* of *P. edulis*, *Zea mays*, and *Populus trichocarpa*, respectively.

Considering that strong purifying selection may shield positive selection at particular amino acid sites or regions, a sliding window analysis was performed to calculate the evolutionary rate for each codon. Most of the *WOX13s* pairs have largely encountered an intense purifying selection (average Ka/Ks = 0.15). Ka/Ks ratios across the coding regions of *OsWOX13_PheWOX13s* and *BradiWOX13_PheWOX13s* were <0.4 (Figure 4a,b), indicating an intense purifying selection across all of the motif positions of WOX13. For gene pairs within species, the majority of the Ka/Ks ratios across the coding regions of *PheWOX13a/b*, *GRMZMWOX13a/b,* and *GRMZMWOX13a/c* were <1, except for the Motif 6 region (Figure 4c,d). A similar status was found at the HD region of *PotriWOX13a_PotriWOX13b* (Figure 4e). Motif 6 of ZmWOX13 and PheWOX13 and the HD of PotriWOX13s might undergo positive selection due to potential redundancy of paralogous function.

**Figure 5:**
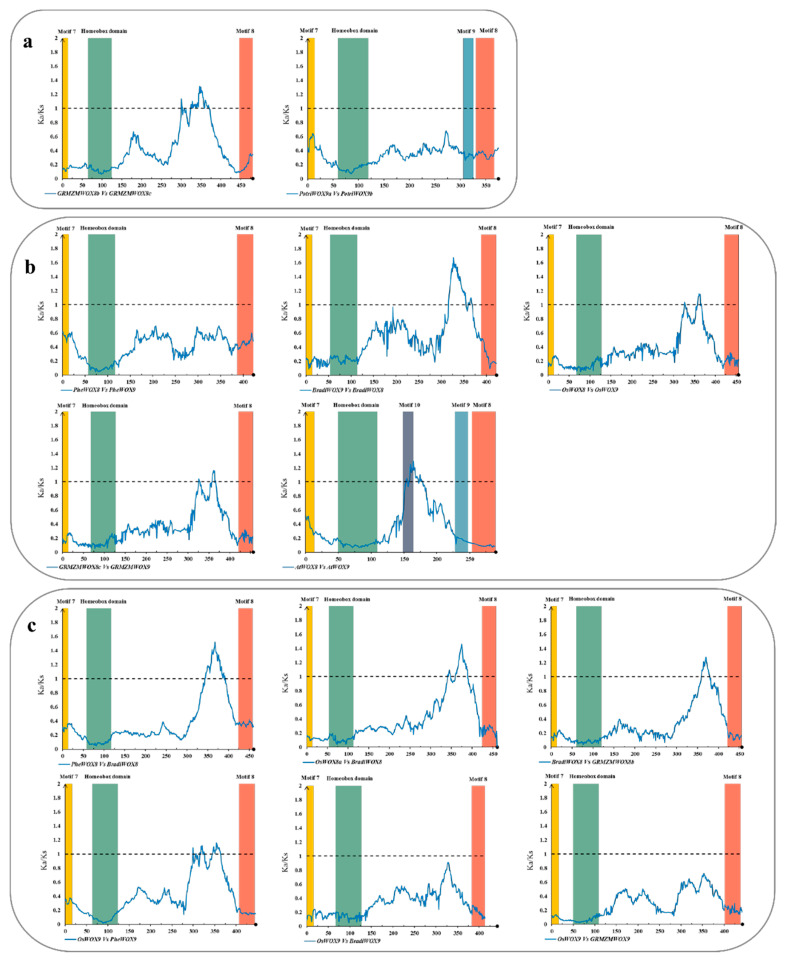
Sliding window of the *WOX8/9* genes of the intermediate clade. (**a**) The sliding window of paralogous genes of *WOX8/9*. (**b**) The sliding window of orthologous genes of *WOX8s*. (**c**) The sliding window of orthologous genes of *WOX9s*.

For the intermediate clade, several sliding window analyses of WOX8/9s and WOX11/12s were performed (Figure 5). Except for the coding region of Motif 8 of *GRMZMWOX11a/b*, it was obvious that the coding regions of Motif 7, the HD, and Motifs 8-10 went through an intensive purifying selection within the intermediate clade (Figure 5 and Supplementary Figure S5). Positive selection was found in the region between the HD and Motif 8 of *GRMZMWOX8a*/*b* (Figure 5a). A similar trace could be found in the *WOX8*/*9* pairs in *Arabidopsis thaliana*, *Oryza sativa*, *Zea mays*, and *Brachypodium distachyon* (Figure 5b), and in the *WOX8* pairs between different species (Figure 5c). The results show a positive selection in the non-conserved region near Motif 8. The coding region upstream of Motif 8 of *WOX8/9* might generate some new function motifs. Particularly, Motif 10, purported to be the specific domain of WOX11/12, was detected with low distinguishability (data not shown) in AtWOX9, while not found in AtWOX8. The alignment region of Motif 10 of *AtWOX9/8* underwent positive selection (Figure 5b).

Members of the WUS clade function as repressors due to the presence of the WUS box. Similar to the specific domain found in other clades, strong purifying selection was found at the region of the HD and the WUS box between/within sub-groups within species, suggesting the importance of the HD and the WUS box for the WUS clade. Positive selection pressure affected the regions between the HD and the WUS box within species, such as *PheWOX2/3a*, *PheWOX3a/b*, *PheWOX4a/b*, *PotriWOX2a/b, GRMZMWOX3a/b, PotriWOX1a/b,* and *AtWOX1/6* (Supplementary Figure S6). Sequence variation was also found outside of the region between the HD and the WUS box in the orthologous genes of different species, such as *OsWOX2_GRMZMWOX2, BradiWOX2 _GRMZMWOX2*, *GRMZMWOX3a_PheWOX3a*, and *AtWOX3_OsWOX3b* (Supplementary Figure S7). These findings evidence the diverse rates of generation of potential functions for members of the WUS clades.

### 3.7. The Expansion of WOXs in Bambusoideae

**Figure 6:**
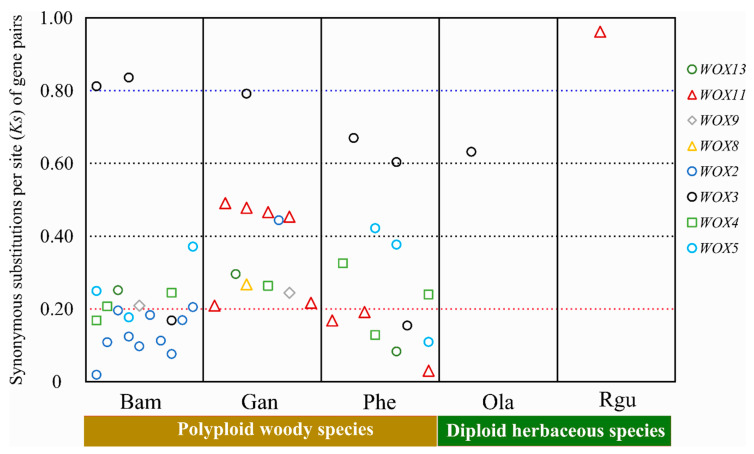
The duplications of the Bambusoideae WOXs of herbaceous and woody bamboos. The colored symbols represent different members of the WOX family. Each symbol indicates a homologous gene pair on the Ks distribution plot. The y-axis indicates the calculated Ks value of each gene pair.

The majority of Bambusoideae *WOXs* underwent strong purifying selection pressure during polyploidization (Supplementary Table S4). Ks value can be used to indicate the divergence time of the duplication event [1,2]. In diploid herbaceous species (*O. latifolia* and *R. guianensis*), two pairs of WOX duplications, namely *OlaWOX3a-OlaWOX3b* and *RguWOX11a-RguWOX11b*, were found with higher Ks versus the majority of WOX duplications in polyploid woody bamboo species (*B. amplexicaulis*, *G. angustifolia*, and *P. edulis*) (Figure 6). *OlaWOX3a-OlaWOX3b* and *RguWOX11a-RguWOX11b* may have occurred by segmental duplications before bamboo speciation. In *B. amplexicaulis, G. angustifolia,* and *P. edulis*, the *WOX3a-WOX3b* pair also showed the high Ks similarity with *O. latifolia* (Figure 6), indicating the divergence of *WOX3a-WOX3b* likely was the result of segmental duplications before the divergence of herbaceous and woody bamboo. In tetraploid woody bamboo species, the duplication pairs of *GanWOX13a-GanWOX13b, GanWOX11a-GanWOX11b, PheWOX11-PheWOX11_like2, GanWOX8a-GanWOX8b, PheWOX8-PheWOX8_like, GanWOX9a-GanWOX9b, PheWOX5a-PheWOX5b/c, GanWOX4a-GanWOX4b,* and *PheWOX4a-PheWOX4c,* that were not tightly cluster in the tree, showed a Ks distance of 0.24~0.49 which indicated a divergence times of 18.4~37.7 Mya (Table S4). The polyploidization events occured 19.7~22.0 Mya which gave rise to the three major lineages, a hexaploid (AABBCC) and two tetraploids (BBCC and CCDD) [1]. These *WOXs* expansion in polyploidy might have resulted from an allopolyploid hybridization. In contrast, the gene pair of *PheWOX13a/b, PheWOX11-PheWOX11-like1, PheWOX12b/c, PheWOX5b/c,* and *PheWOX3b1/b2* were cluster tightly, and showed a divergence times of 2.4~11.9 Mya (Table S4), which very likely resulted from the segmental DNA duplications. In *B. amplexicaulis*, more duplications of *WOX2* were found with a narrow Ks distance of 0.02-0.21, suggesting frequent segmental duplications of WOX2 in recent times.

### 3.8. The Similar cis-Element Positions of the Orthologous WOX Promoters in Bambusoideae

**Figure 7:**
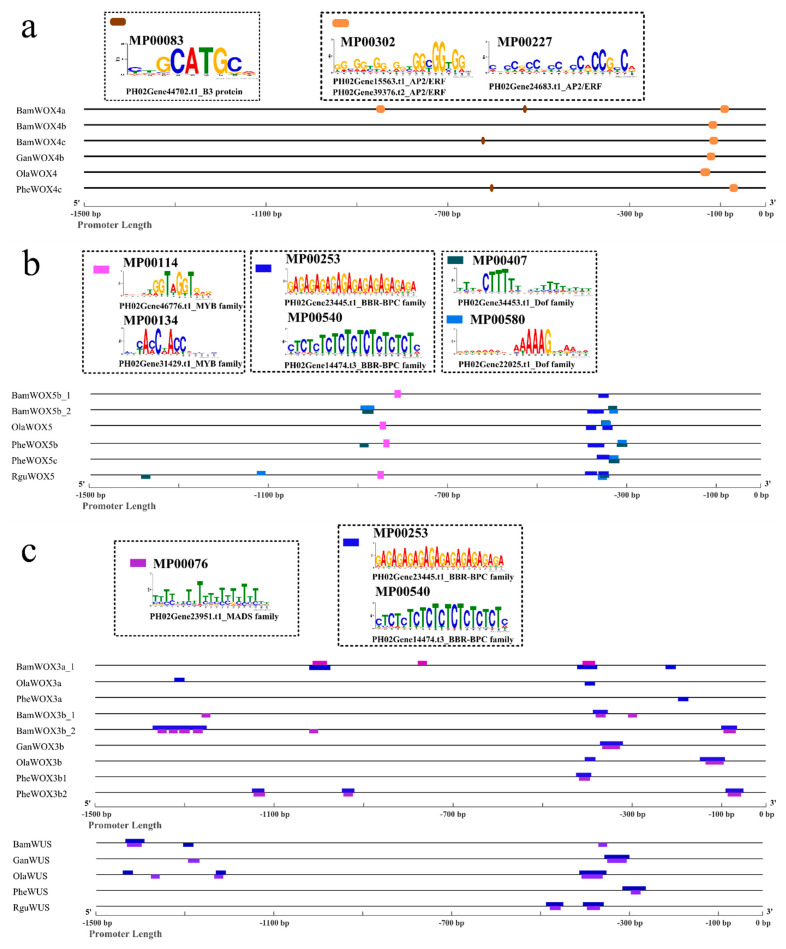
The conserved cis-element distribution in the promoters of Bambusoideae *WOX4* (**a**), *WOX5* (**b**), and *WOX3* and *WUS* (**c**). The 1500-bp-long promoters were analyzed by PlantRegMap. The ID of the cis-elements was in accordance with the transcription factor (TF) binding motif dataset of PlantTFDB and is shown with different colored rectangles. The TFs were predicted by comparing homologous genes with TF datasets of Arabidopsis (http://planttfdb.cbi.pku.edu.cn/index.php?sp=Ath).

To evaluate potential upstream regions for potential common cis-regulatory sequences, we scanned regions 1500 bp upstream of the start codons for TF-binding motifs using PlantRegMap. TF-binding sites in the *WOX* promoters were identified based on the TF-binding motif dataset of PlantRegMap (Supplementary Table S5). The promoters of *BamWOX4a/b/c*, *PheWOX4c*, *GanWOX4b*, and *OlaWOX4* were clustered in same branch (Supplementary Figure S7). MP00302/MP00227 was found generally at around the −100 bp position of these promoters (Figure 7a), but not found in the promoters of *OsWOX4* or *AtWOX4* (Supplementary Table S5). The B3 protein binding element, MP00083, was found in a similar region as that of the promoters of *BamWOX4a/c* and *PheWOX4b/c* (Figure 7a, Supplementary Table S5). MP00265 (CA[T/A]TCA[T/A]TCA), recognized by WUS, was found in the promoters of *PheWOX4b* (−970~−959 bp) and *OsWOX4* (−1278~−1268 bp), while not found in the promoters of *AtWOX4* or the other bamboo *WOX4s* (Supplementary Table S5). The promoters of *PheWOX5b/c*, *BamWOX5b1/b2*, *RguWOX5*, and *OlaWOX5* clustered in same branch (Supplementary Figure S7). Two conserved positions were found in these promoters, namely, −400~−300 bp and −900~−800 bp upstream from the initiation codon (Figure 7b). At the position of −400~−300 bp, two consistent cis-elements, namely, MP00407/MP00580 and MP00253/MP00540, were found. MP00407 was also found in the promoters of *AtWOX5* and *OsWOX5*. MP00114/MP00134 was scanned at the position of −900~−800 bp. The Bambusoideae WUS and WOX3a/b were clustered in three separate branches with high support (Supplementary Figure S7). A GA-rich element (MP00253/MP00540) was also found conserved in the promoters of bamboo and rice WOX3/WUS (Figure 7c). A MADS-binding sites (MP00076) was found in most *WOX3* and *WUS* promoters and was predicted to be the target of PH02Gene23951.t1, homology to *OsMADS56* of rice (Figure 7c). *OsMADS56* regulates rice flowering antagonistic with *OsMADS50* [47]. The promoters of *BamWOX8*, *GanWOX8a,* and *OlaWOX8* showed two similar cis-elements, namely, MP00411/MP00302/MP00227 and MP00513/MP00249 (Supplementary Figure S8a). MP00076 was found at the promoters of *BamWOX9b*, *GanWOX9b*, *OlaWOX9,* and *PheWOX9*. An MYB-binding site (MP00513/MP00249) was also found in these promoters, 70 bp away from MP00076. MP00513/MP00249 was also found in the promoters of *AtWOX9* and *OsWOX9*. At 1000 bp upstream from the start codon, abundant AP2/ERF-binding sites were found in the *BamWOX9b*, *OlaWOX9,* and *PheWOX9* promoters (Supplementary Figure S8b). These sites, found approximately positioned in the promoters of bamboos’ orthologous *WOXs,* may play an important role in regulating the expression of *WOXs*.

## 4. Discussion

### 4.1. Conserved Domains were Retained during Species Differentiation

The evolution of the WOX family played pivotal roles in morphological innovations during the evolutionary history of species differentiation in the plant kingdom [8,43,44,48,49,50,51,52]. The large-scale identification of the WOX family from Viridiplantae has been extensively studied, including species of lower and higher plants [42]. The ancient clade comprises WOX10/13/14 proteins from charophytes, bryophytes, gymnosperms, lycophytes, ferns, and angiosperms. The intermediate clade contains WOX8/9 and WOX 11/12 proteins from gymnosperms and angiosperms. The WUS clade includes WUS, WOX5/7, WOX3, WOX1/6, WOX4, and WOX2 proteins from gymnosperms and angiosperms, and some other WOX proteins of lycophytes and ferns that cluster into the branch that separates the subclades of gymnosperms and angiosperms [42]. It is clear that *WOXs* are ubiquitous in most plants, except for rhodophytes, and that members of the major clades were formed alongside plant evolution.

In addition to HDs, which are present as DNA-binding domains in all of the WOX members [9,40,42,53], ten specific domains of three major WOX clades were identified in this study. Similar positions and high conservatism could be observed from the evolutionary topology (Figure 2). Motif 4 is found in the N-terminal domain of most WOX proteins in the ancient clade [9,40,42]. Motif 5 is found in the C-terminal of most WOX13 proteins, but absent in WOX10/14 proteins [9]. Moreover, a new specific domain, Motif 6, was identified in the C-terminal of WOX13 in rice, maize, *Brachypodium distachyon*, and five bamboo species (Figure 2 and Figure 3). A species-wide analysis of Motif 6 was performed in the WOX13s of monocots and dicots, where Motif 6 was found in the WOX13s of 86.8% of monocots (33 of 38 monocots) and 32.0% dicots (32 of 100 eudicots, in which 24 species belong to asterids) (data not shown). This suggests that the conservation of Motif 6 shows species specificity. Motifs 8–9 (named T2WOX) have been reported as specific domains of the intermediate clade in research of the WOX family from 267 species [42]. However, Motif 9 cannot be observed in WOX8/9s of rice, maize, *Brachypodium distachyon*, or five bamboo species. As per previous reports, Motif 10 is a typical domain found in WOX11/12s [9]. Motif 10 (the WUS box) can be found in the majority of the members of the WUS clade [9,12,42,46,54,55]. Moreover, an EAR-like motif, L(D/E)L(R/S)L, was found in WUS and WOX5/7 proteins [9,42], but not found in the other subclade of the WUS clade.

### 4.2. The Evolution of the WUS Clade was Likely Selected by its Conserved and Essential Function in Plants

The WOX family regulates key plant developmental processes, especially for organ morphogenesis. WOXs have essential functions in cell population identification and differentiation, in regulating stem cell identity in SAM and root apical meristems (RAM) and vascular cambium [11,12,56,57,58], in polarity pattern formation [21], and in the development of lateral organs [59,60]. Members of the WOX family are highly conserved, indicating their similar functions among species, which is reflected in the conserved motif found in each clade among the different species.

The functions of AtWUS and AtWOX5 are complementary to each other in the SAM and RAM of Arabidopsis [12,57] via a similar regulative signaling pathway [56,61,62]. Null mutations of Arabidopsis *PRESSED FLOWER1* (*PRS1*, *AtWOX3*) prevent the initialization of lateral organs and cause the preprimordial deletion of lateral stipules from vegetative leaves and lateral sepals/stamens of flowers [51], which can be rescued by *AtWUS* and *AtWOX4* when driven by the PRS1 native promoter [19,55]. Null mutations of WOX3s cause narrow leaf phenotypes in some monocots, like maize [49], rice [63], and barley [20], which are similar to the WOX1 mutants of some dicots, like *Petunia* (*maw*) [60], *Medicago truncatula* (*stf*) [64], and *Nicotiana sylvestris* (*lam1*) [65]. All members of the Arabidopsis WUS clade, except for WOX7 (loss of the WUS box, Figure 2), could complement *lam1* mutation when driven by the *stf* native promoter of *Medicago truncatula* [46]. The members of the intermediate and ancient clades driven by the *stf* promoter cannot complement *lam1* mutation unless a supererogatory repressor domain adheres [46], indicating the importance of the WUS box as transcriptional repressor domain. Intensive purifying selection was found in the coding regions of both the HD and the WUS box domains among species by sliding window analysis (Supplementary Figures S6 and S7), confirming the importance of the presence of the HD and the WUS box for the conserved function of the WUS clade. WOX genes of WUS clade are functionally redundant, and could replace each other if expressed in the necessary developmental time and space. It seems that the subfunctionalization of the WUS clade genes most likely depends on the diverse promoter specificity of paralogous genes. In bamboos, conserved evolution of Bambusoideae *WOX4s* and *WOX5s* was found at the transcriptional and translational levels, as well as cis-element distribution in the promoter region, which indicates the important function of *WOX4s* and *WOX5s* for bamboo development.

### 4.3. The Conserved cis-Element Distribution of Bambusoideae WOXs May Relate to Their Functions during Development

In our study, some particular cis-elements found at the same positions of orthologous genes of different bamboos may regulate bamboo development indispensability. A WUS binding site was found in the promoters of *PheWOX4a* and *OsWOX4*. In rice, *OsWUS* was expressed in the premeristem zone in the early stage, and then disappeared in the axillary meristem after its establishment. However, *OsWOX4* was expressed in the established axillary meristem, but either no or very weak expression was detected in the early premeristem zone [17]. MP00265 might be related to the switch between *OsWUS* and *OsWOX4* found during axillary formation in rice.

MP00253/MP00540 were found at the same positions of the *WOX5*, *WOX3*, and *WUS* promoters, which are GA-rich elements (GAGA-repeats) present in the regulatory sequences of genes involved in developmental processes [66,67]. MP00407/MP00580, found in the *WOX5* promoter, showed a core sequence of 5’-AA[AG]G-3’ recognized by two PheDofs (PH02Gene34453.t1 and PH02Gene22025.t1), homologous to AtDOF5.6 (also named as HCA2) and AtDOF3.6 (also named as OBP3) of *Arabidopsis thaliana*. Two positions of MP00407 were found at the regions of −613~−593 bp and −967~−947 bp in the promoter of *AtWOX5*, and found at the position of −927~−907 bp in the promoter of *OsWOX5* (Supplementary Table S5). *HCA2* expressed in the vascular tissues and pericycle of primary roots has played a vital role in the promotion of both cambium activity and phloem specification [68]. *OBP3* has been reported as a salicylic acid-induced gene predominantly expressed in roots [69], and overexpression of *OBP3* results in defective roots [70]. Another site in the *WOX5* promoter, MP00114/MP00134, was recognized by PH02Gene31429.t1 and PH02Gene46776.t1, homologous to AT1G09540.1 (AtMYB61) and AT3G61250.1 (AtMYB17, LMI2), respectively. *AtMYB61* coordinates the target genes required for xylem formation, xylem cell differentiation, lateral root formation, etc. [71]. *AtMYB17* mainly functions in the transition of meristem identity from vegetative growth to flowering [72]. The −400 bp~−300 bp and −900~−800 bp regions of the bamboo *WOX5* promoter may play a vital role in regulating the expression of *WOX5* during bamboo adventitious root formation.

MP00076 is found in the majority of bamboo *WOX3* and *WUS* promoters. We investigated the *AtWOX3* promoter, and also found MP00076 in the region of −662 to −638 bp and −1038 to −1018 bp. *AtSOC1* (AT2G45660.1) could bind to MP00076, and promote inflorescence meristem identity during the transition to flowering of *Arabidopsis thaliana* [73]. MP00076 was also found in the promoters of *BamWOX9b*, *GanWOX9b*, *OlaWOX9*, and *PheWOX9*. *WOX3*, *WUS,* and *WOX9* have been observed to regulate floral development [74]. We speculate that bamboo WUS, WOX3, and WOX9 may mediate meristem floral development and architecture, similar to previous studies.

## 5. Conclusions

In this study, 12 *OlaWOXs*, 9 *RguWOX,* 18 *GanWOXs*, 27 *PheWOXs*, and 26 *BamWOXs* were identified and categorized into ancient, intermediate, and WUS clades. Except for a universal HD found in all WOXs, eight specific domains were identified—three found in the ancient clade, four found in the intermediate clade, and one (the WUS box) found in the WUS clade. Intensive purifying selection in the coding region of specific domains leads to the conserved function of orthologous genes. For Bambusoideae *WOXs*, polyploidy is the major driver of the expansion of the *WOX* family. A low evolutionary rate of orthologous genes was found within the WOX family of Bambusoideae. AP2/ERF protein-binding regions and B3 protein-binding regions were found at the similar position of bamboo *WOX4* promoters. Moreover, MYB protein-binding regions and Dof protein-binding regions were also discovered in the promoters of bamboo *WOX5*. These conserved *cis*-elements may play an important role in regulating the expression of *WOX4* and *WOX5*. Flowering-related MADS binding sites were found in the promoters of bamboo *WOX3*, *WUS,* and *WOX9*, which indicate their potential roles in regulating bamboo flower formation.

## Figures and Tables

**Table 1:** Comparison of the *WOX*s of Bambusoideae with other species.

Taxonomic Group	Species	Ancient Clade	Intermediate Clade	WUSCHEL (WUS) Clade	Other	Total
Dicots	*Arabidopsis thaliana*	3	4	8	-	15
	*Populus trichocarpa*	3	4	11	-	18
Monocots	*Oryza sativa*	1	6	6	-	13
	*Brachypodium distachyon*	2	5	6	-	13
	*Zea mays*	3	7	10	-	20
	*Olyra latifolia*	1	5	6	-	12
	*Raddia guianensis*	1	3	5	-	9
	*Guadua angustifolia*	2	9	7	-	18
	*Phyllostachys edulis*	2	11	14	-	27
	*Bonia amplexicaulis*	2	6	18	-	26
Chlorophyta	*Micromonas pusilla*	-	-	-	4	4
	*Micromonas sp.*	-	-	-	1	1
	*Ostreococcus tauri,*	-	-	-	1	1
	*Bathycoccus prasinos*	-	-	-	1	1

## Supplementary Materials

The following are available online at https://www.mdpi.com/2218-273X/10/5/739/s1, Figure S1: The Maximum Likelihood tree of WOXs from dicot and monocot; Figure S2: The alignment of WOX13s amino acid sequences; Figure S3: The alignment of the genome sequences of Bambusoideae WOX4s; Figure S4: The alignment of the genome sequences of Bambusoideae WOX5s; Figure S5: Sliding-window of the WOX11/12s; Figure S6 Sliding-window of the paralogous genes in the WUS clade; Figure S7: Sliding-window of the orthologous genes in the WUS clade; Figure S8: The conservative cis-element distribution in the promoters of Bambusoideae WOX8(a), WOX9(b), Tables S1: The informations of Bambusoideae WOXs; Tables S2: The informations of dicot and monocot WOXs used in this study; Table S3: The Ka/Ks ratio of dicot and monocot WOXs in sub-group; Tables S4: The Ka/Ks ratio of Bambusoideae WOXs in sub-group; Table S5: The cis-elements predicted in the promoter of Bambusoideae WOXs, Dataset S1: Polypeptides used to construct phylogenetic tree of AtWOXs, PotriWOXs, OsWOXs, ZmWOXs, BradiWOXs, and PheWOXs.
