# Supplementary material for: Identification and Evolution of the WUSCHEL-Related Homeobox Protein Family in Bambusoideae
Source: Biomolecules. 2020 May 9;10(5):739. doi: 10.3390/biom10050739 (PMC7278010; doi:10.3390/biom10050739)
Supplement: Supplementary file 1 [file biomolecules-10-00739-s001.zip › biomolecules-742176 - for proof supple/Supplemental Figures.docx]

**Supplemental Figure legends**

**Figure S1.** The Maximum Likelihood tree of WOXs from dicot and monocot. Leaf colors of green, orange, and cyan stand for WOXs of chlorophytes, monocots, and dicots, respectively.

**Figure S2.** The alignment of WOX13s amino acid sequences.

**Figure S3.** The alignment of the genome sequences of Bambusoideae *WOX4s*.

**Figure S4.** The alignment of the genome sequences of Bambusoideae *WOX5s*.

**Figure S5.** Sliding-window of the *WOX11/12s*.

**Figure S6.** Sliding-window of the paralogous genes in the WUS clade.

**Figure S7.** Sliding-window of the orthologous genes in the WUS clade.

**Figure S8.** The conservative cis-element distribution in the promoters of Bambusoideae *WOX8*(a), *WOX9*(b).

**Supplementary Dataset legends**

**Supplementary Dataset S1.** Polypeptides used to construct the phylogenetic tree of AtWOXs, PotriWOXs, OsWOXs, ZmWOXs, BradiWOXs, and PheWOXs.

**Supplemental Tables legends**

**Supplemental Tables S1.** The informations of Bambusoideae *WOXs*.

**Supplemental Tables S2.** The informations of dicot and monocot *WOXs* used in this study.

**Supplemental Tables S3.** The Ka/Ks ratio of dicot and monocot *WOXs* in sub-group.

**Supplemental Tables S4.** The Ka/Ks ratio of Bambusoideae *WOXs* in sub-group.

**Supplemental Tables S5.** The *cis*-elements predicted in the promoter of Bambusoideae *WOXs*.

**
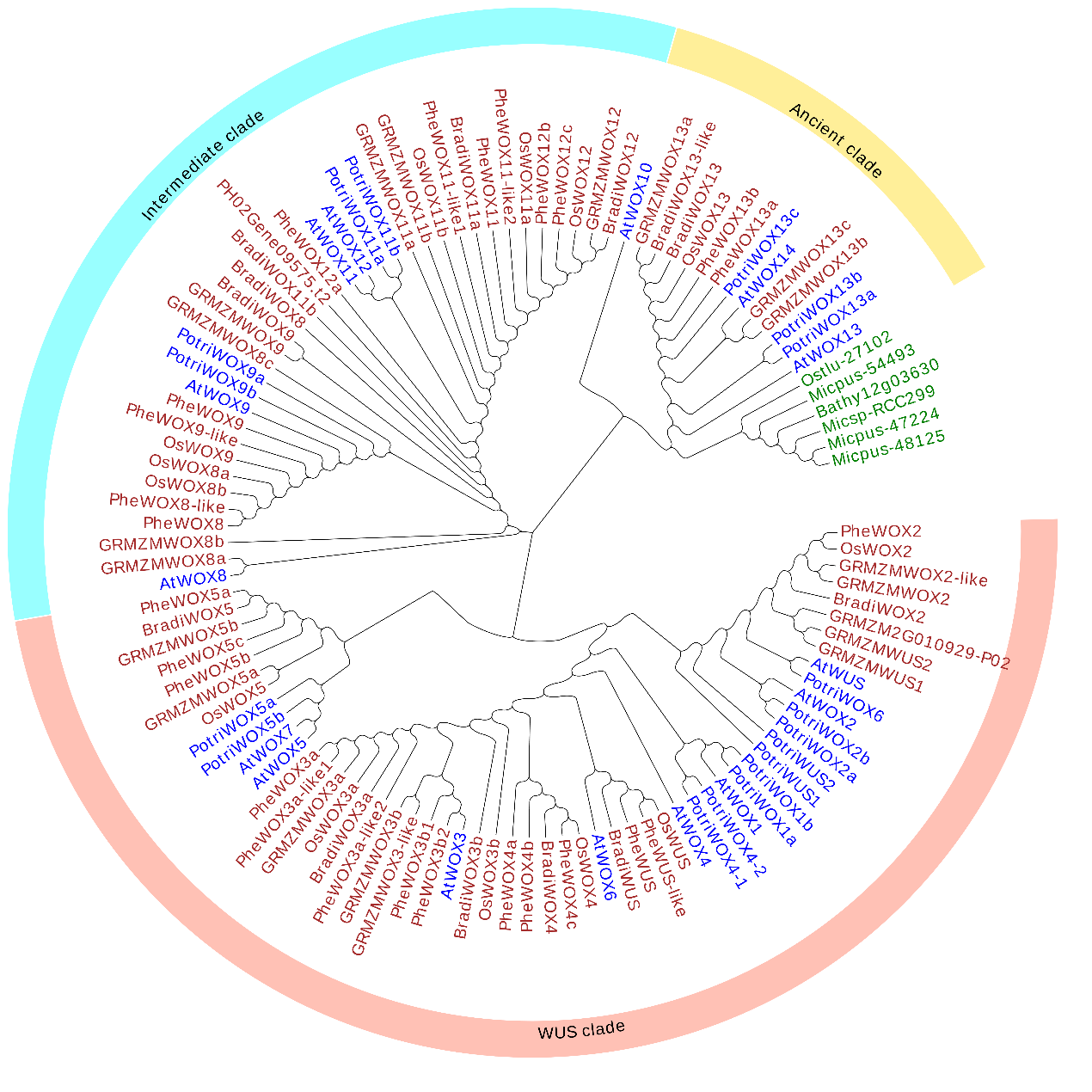
**

**Figure S1**

**
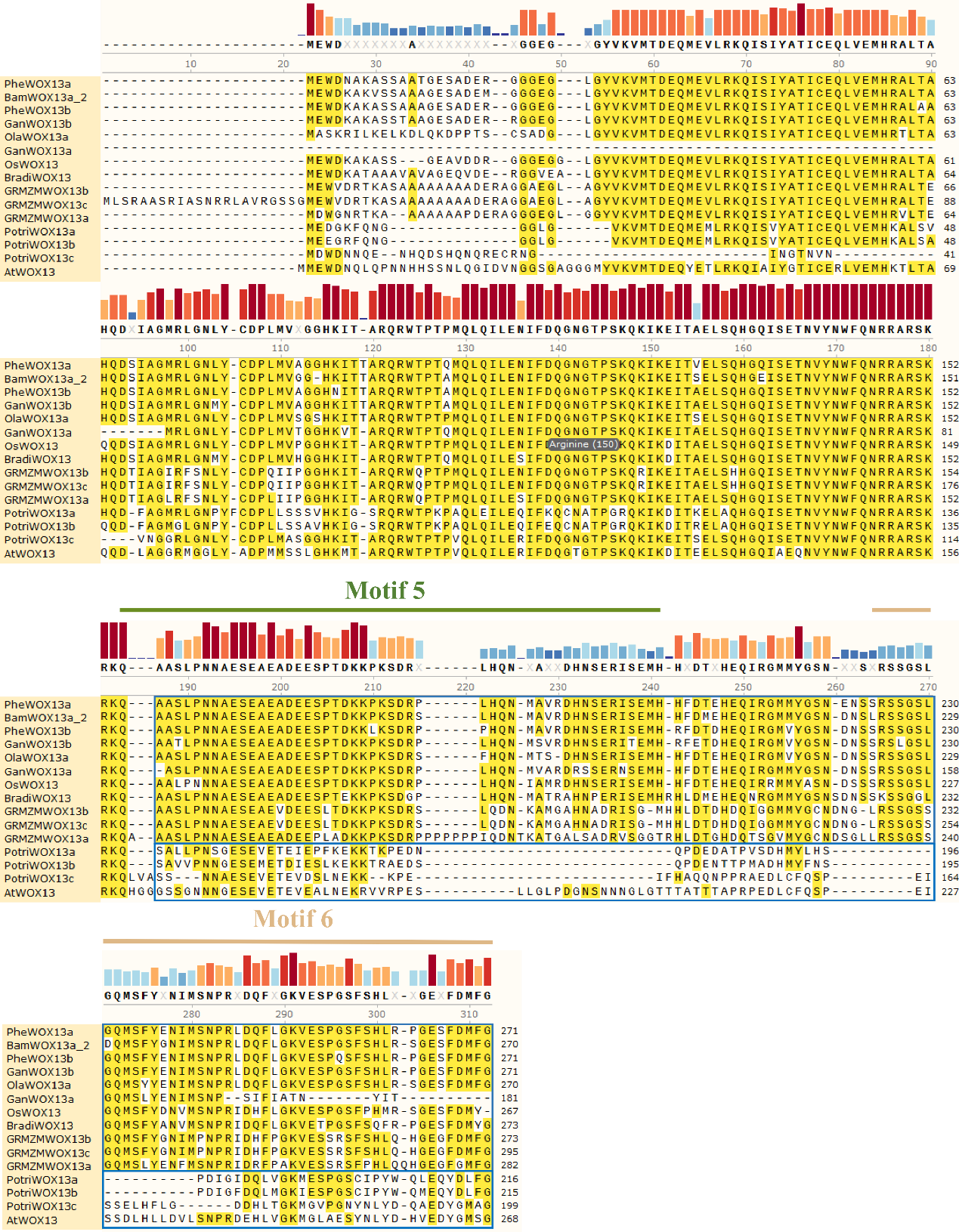
**

**Figure S2**

**
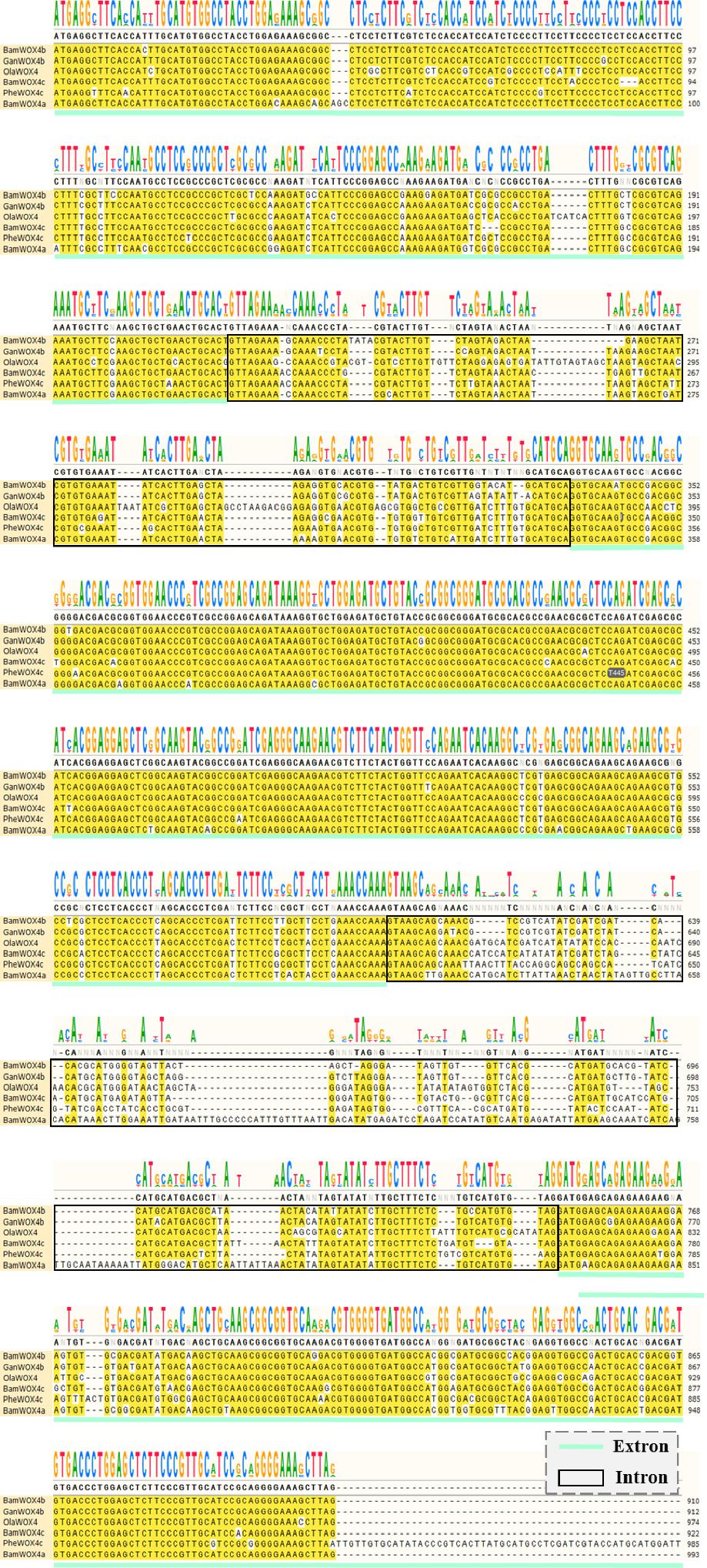
Figure S3**

**
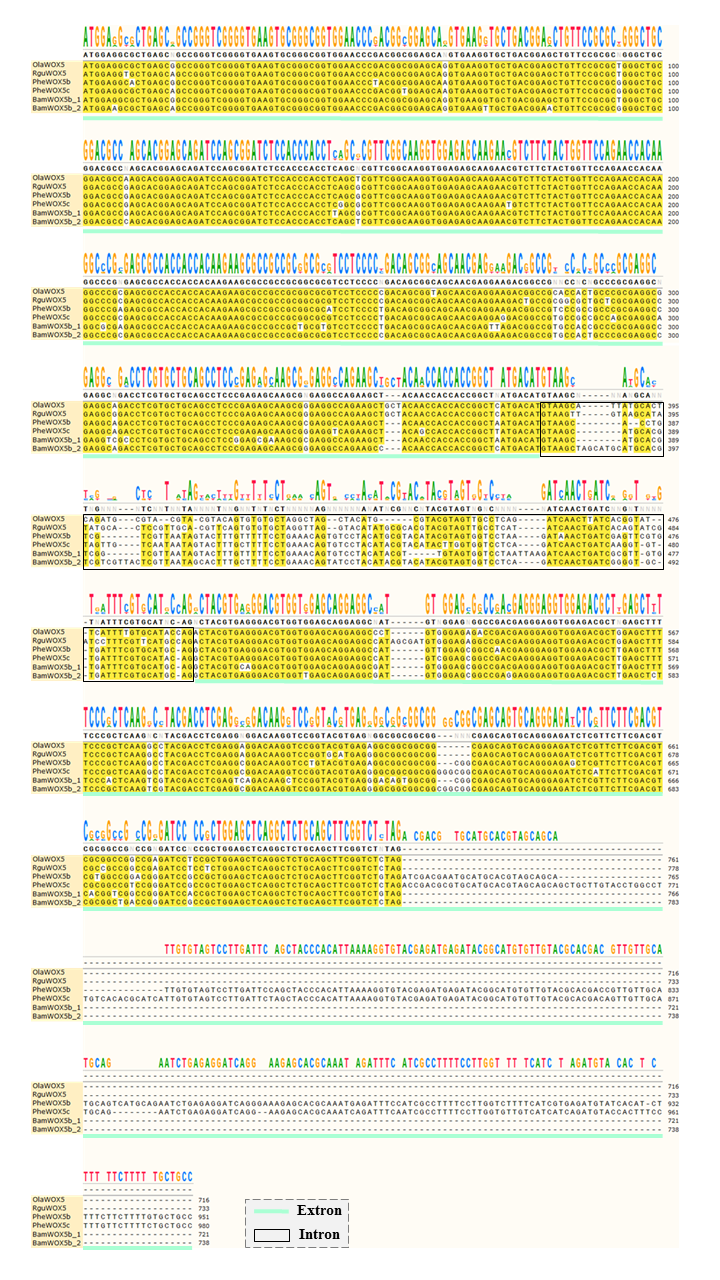
**

**Figure S4**

**
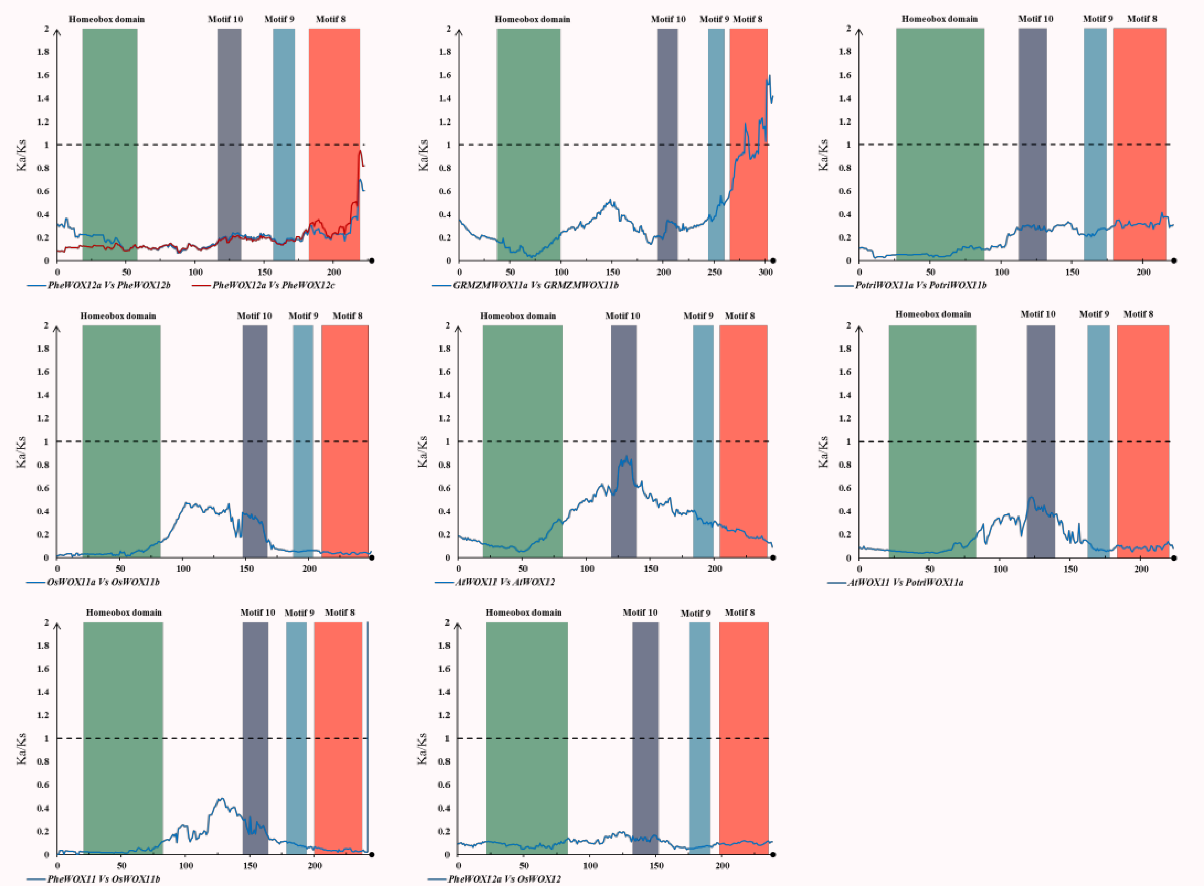
**

**Figure S5**

**
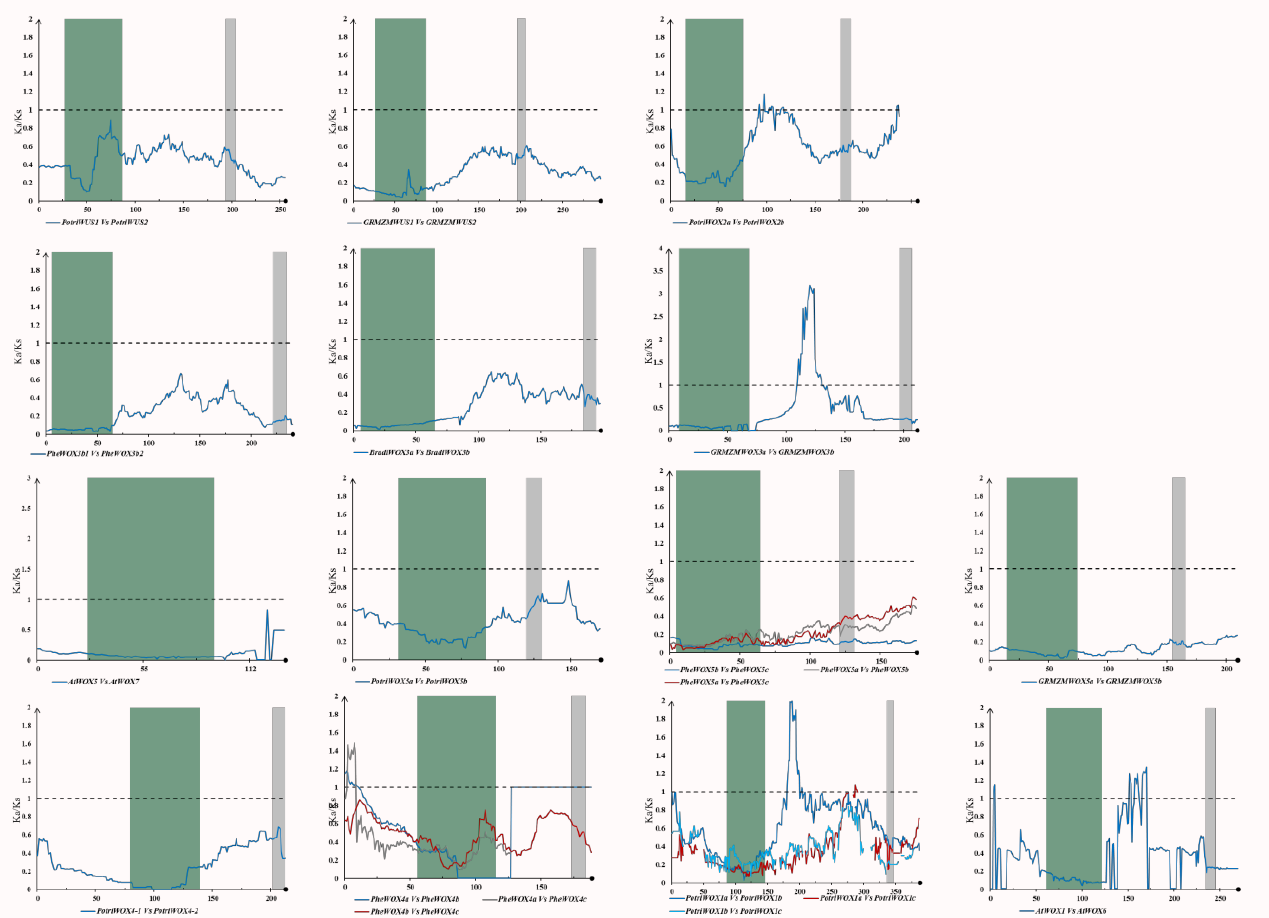
**

**Figure S6**

**
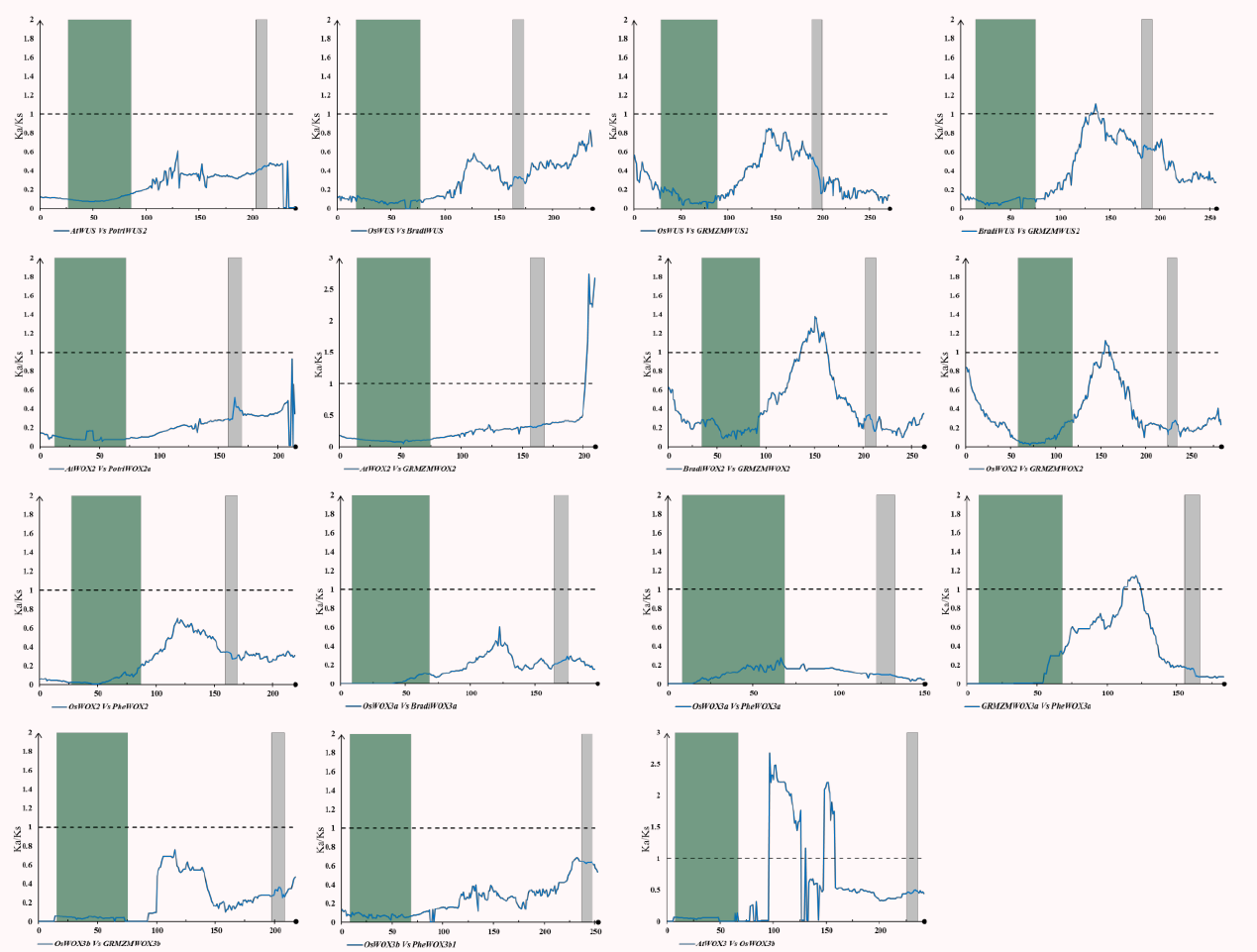
**

**Figure S7**

**
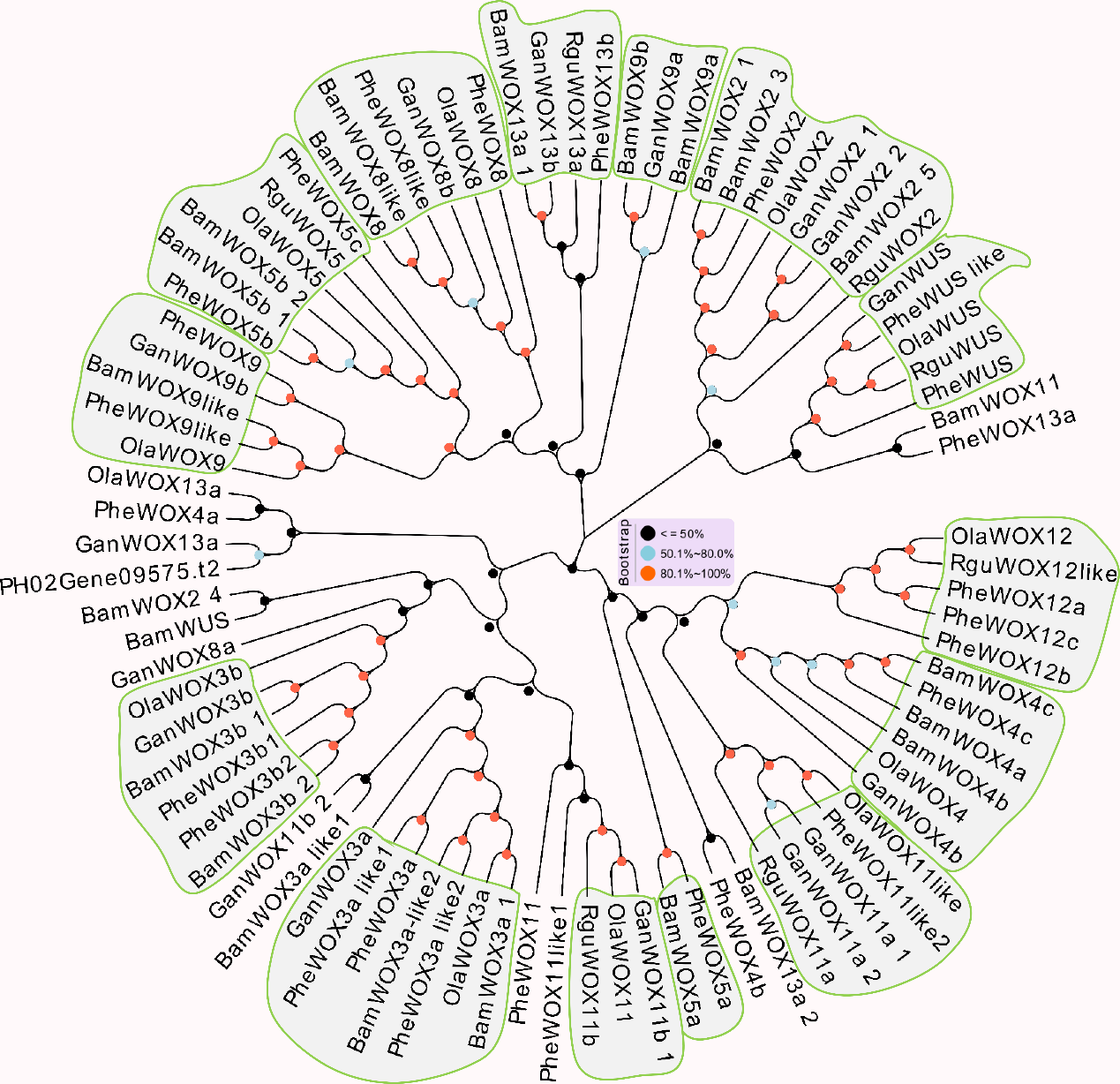
**

**Figure S8**

**
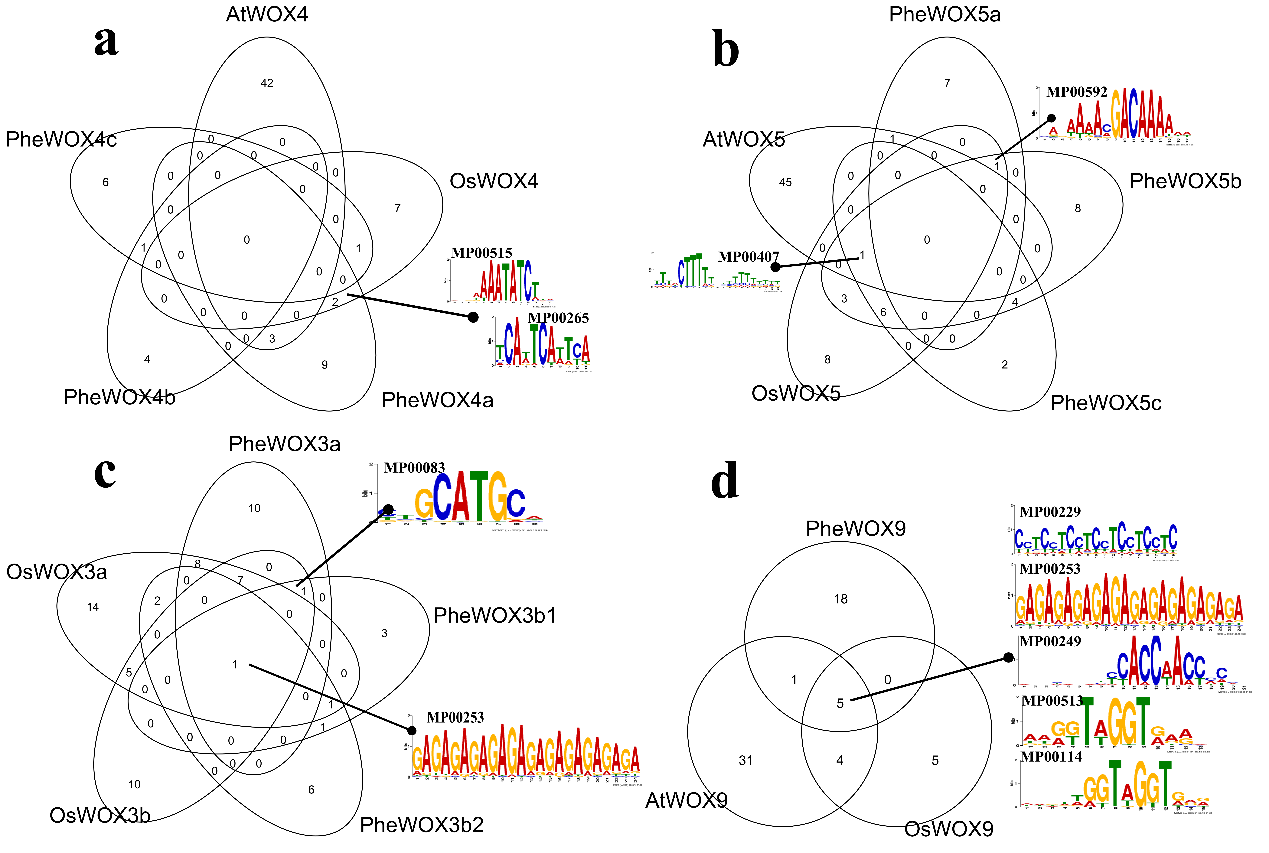
 Figure S9**


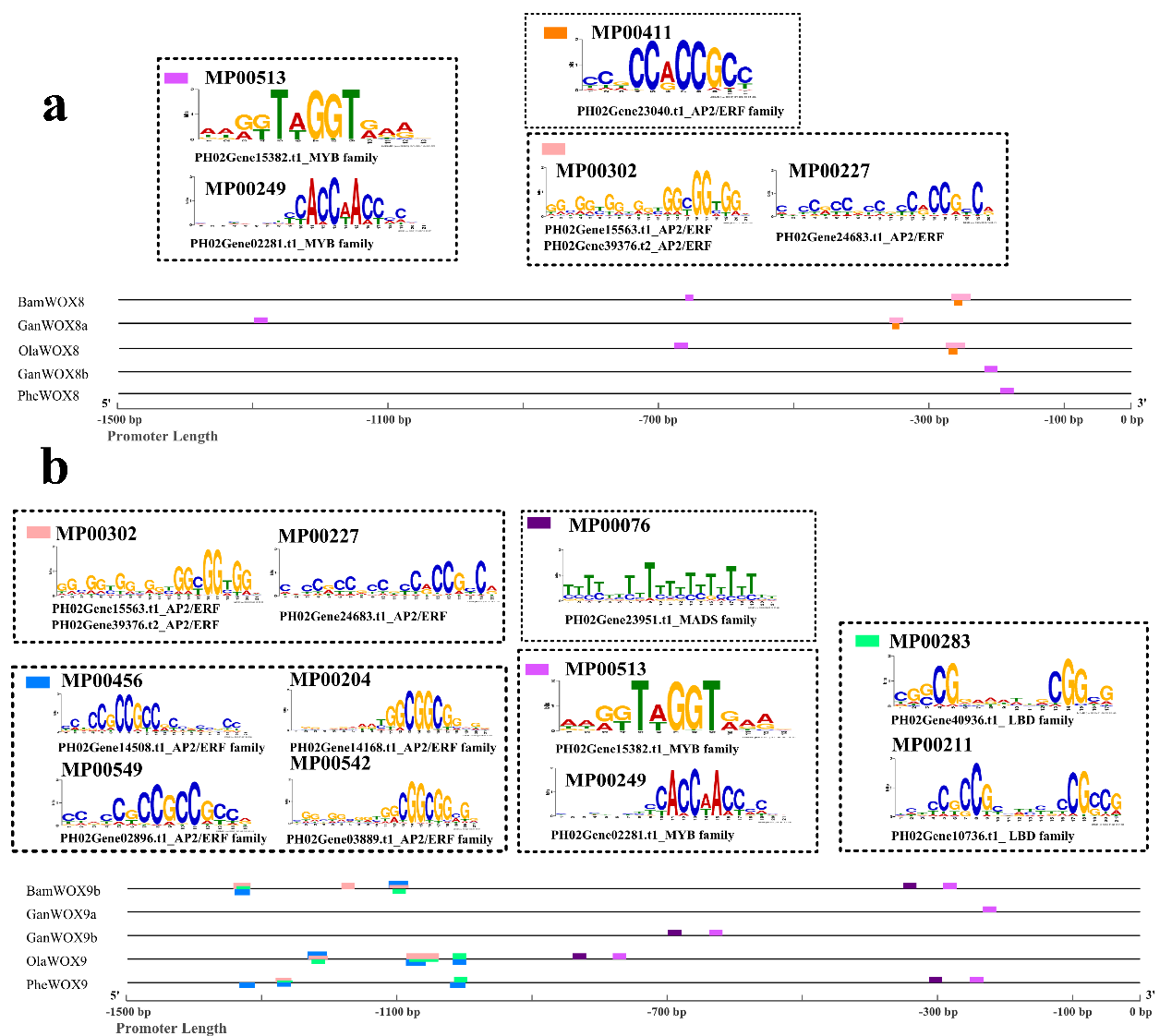


**Figure S10**
