# Supplementary material for: Identification and Evolution of the WUSCHEL-Related Homeobox Protein Family in Bambusoideae
Source: Biomolecules. 2020 May 9;10(5):739. doi: 10.3390/biom10050739 (PMC7278010; doi:10.3390/biom10050739)
Supplement: Supplementary file 1 [file biomolecules-10-00739-s001.zip › biomolecules-742176 - for proof supple/Supplementary Dataset S1.docx]

**Supplementary Dataset S1.**

**Polypeptides used to construct phylogenetic tree of AtWOXs, PotriWOXs, OsWOXs, ZmWOXs, BradiWOXs, and PheWOXs.**

>Micpus-48125

PRGPSAPRWNPTPAQLARLEELFLTGMGTPNGELRTQITEELAKLGPINEANVYNWFQNKKARMKKAEREREAALAAGRPPPV

>Micpus-47224

LAINALGVNGEIIQTTTLIVKGVSPQGPSARANSLVSRPHLERSARWNPTPAQLARLEELFLTGMGTPKREQRTQITEELAKLGPINEANVFNWFKNKKSKMKRDASQKGGHEKENASSRVFTPRDYTLLELMQRKPRVNDDDADASFRS

>Micsp-RCC299

PRGARWTPSAAQLARLEELFATGMGTPNGDLRTKITDELAKLGPVNEANVYNWFQNKKARTKKKLLEEQAAKNRSG

>Bathy12g03630

WSDGLYPDPRTLTLISVFHKNHRAENSSQKHLISIHHRPHNLKRVWKTRETFHHAMSSLAEQPPQAAAQEGAVDTTITTAADAAEKNKNDDDVEMKEANDNIIITTTAPAAPPSAAAAEIDDGAAADEEEEDAGGEKNEQEEKEEKEQEEEEEREKYDPKEGPKILANVPDLPRNAALHMTEQQKYALRQQIASYAHICQQLLQLTHEAALRDLQMSARGEYDRHATHASSAYPREKQPKKEKPHKERKTPASIFSNTGVYSVPGKVRWQPTTAQFERLEQLFAIDTTTPQRENLKQVTEELSALGPIQECNVYNWFQNKKARLKKREQDAARERMEEEMRAKADQYNAQQQQMGNLS

>Micpus-54493

PCGGDVFGGRATRPMNYSDPWSVNDAGESDQESGFLSSLVGVGEANATTATKTKPAELPETSPPPAPRWRATPEQRARLDELFETDDAVPKEERKSEITRELRAFGPIEERNVHFWFANRRREKKKKARDAEAAAAGAGVGGTAGEAVDAPIEIDD

>Ostlu-27102

KGVRGPRWSGTPDQYKILEDLFLAGEQPPVRARLTEITKRLQEHGPIQEHNVYNWFQNRRSREKKRLAEERASNDAALDNQPLASRYGVGER

>AtWOX10

TRPRWTPTTTQLQILENIYKEGSGTPNPRRIKEITMELSEHGQIMEKNVYHWFQNRRARSKRKQPPTTTITSSQADDAAVTTTEERGRCGDDSGGFESYEHILFPSPDLGIEHLLNRDKFID

>AtWOX14

TRHRWTPTSTQLQILESIYDEGSGTPNRRRIREIATELSEHGQITETNVYNWFQNRRARSKRKQPQTTTANGQADDVAVTTEERRSCGDSGGLESYEHILFPSPDLGIEHLLSIGKFMET

>AtWOX13

ARQRWTPTPVQLQILERIFDQGTGTPSKQKIKDITEELSQHGQIAEQNVYNWFQNRRARSKRKQHGGGSSGNNNGESEVETEVEALNEKRVVRPESLLGLPDGNSNNNGLGTTTATTTAPRPEDLCFQSPEISSDLHLLDVLSNPRDEHLVGKMGLAESYNLYDHVEDYGMSG

>PotriWOX13c

ARQRWTPTPVQLQILERIFDQGNGTPSKQKIKEITSELSQHGQISETNVYNWFQNRRARSKRKQLVASSNNAESEVETEVDSLNEKKKPEIFHAQQNPPRAEDLCFQSPEISSELHFLGDDHLTGKMGVPGNYNLYDQAEDYGMAG

>PheWOX13a

ARQRWTPTQMQLQILENIFDQGNGTPSKQKIKEITVELSQHGQISETNVYNWFQNRRARSKRKQAASLPNNAESEAEADEESPTDKKPKSDRPLHQNMAVRDHNSERISEMHHFDTEHEQIRGMMYGSNENSSRSSGSLGQMSFYENIMSNPRLDQFLGKVESPGSFSHLRPGESFDMFG

>PheWOX13b

ARQRWTPTAMQLQILENIFDQGNGTPSKQKIKEITAELSQHGQISETNVYNWFQNRRARSKRKQAASLPNNAESEAEADEESPTDKKLKSDRPPHQNMAVRDHNSERISEMHRFDTDHEQIRGMVYGSNDNSSRSSGSLGQMSFYENIMSNPRLDQFLGKVESPQSFSHLRPGESFDMFG

>OsWOX13

ARQRWTPTPMQLQILENIFDQGNGTPSKQKIKDITAELSQHGQISETNVYNWFQNRRARSKRKQAALPNNNAESEAEADEESPTDKKPKSDRPLHQNIAMRDHNSERISEMHHFDTEHEQIRRMMYASNDSSSRSSGSLGQMSFYDNVMSNPRIDHFLGKVESPGSFPHMRSGESFDMY

>BradiWOX13

ARQRWTPTQMQLQILESIFDQGNGTPSKQKIKDITAELSQHGQISETNVYNWFQNRRARSKRKQAASLPNNAESEAEADEESPTEKKPKSDGPLHQNMATRAHNPERISEMHRHLDMEHEQNRGMMYGSNSDNSSKSSGGLGQMSFYANVMSNPRIDQFLGKVETPGSFSQFRPGESFDMYG

>BradiWOX13like

ARQRWTPTQMQLQILESIFDQGNGTPSKQKIKDITAELSQHGQIETNVYNWFQNRRARSKRKQAAFLPNNT

>GRMZMWOX13b

ARQRWQPTPMQLQILENIFDQGNGTPSKQRIKEITAELSHHGQISETNVYNWFQNRRARSKRKQAASLPNNAESEAEVDEESLTDKKPKSDRSLQDNKAMGAHNADRISGMHHLDTDHDQIGGMMYGCNDNGLRSSGSSGQMSFYGNIMPNPRIDHFPGKVESSRSFSHLQHGEGFDMFG

>GRMZMWOX13c

ARQRWQPTPMQLQILENIFDQGNGTPSKQRIKEITAELSHHGQISETNVYNWFQNRRARSKRKQAASLPNNAESEAEVDEESLTDKKPKSDRSLQDNKAMGAHNADRISGMHHLDTDHDQIGGMMYGCNDNGLRSSGSSGQMSFYGNIMPNPRIDHFPGKVESSRSFSHLQHGEGFDMFG

>GRMZMWOX13a

ARQRWQPTPMQLQILESIFDQGNGTPSKQKIKEITAELSQHGQISETNVYNWFQNRRARSKRKQAAASLPNNAESEAEADEEPLADKKPKSDRPPPPPPPIQDNTKATGALSADRVSGGTRHLDTGHDQTSGVMYGCNDSGLLRSSGSSGQMSLYENFMSNPRIDRFPAKVESSRSFPHLQQHGEGFGMFG

>PotriWOX13a

SRQRWTPKPAQLEILEQIFKQCNATPGRQKIKDITKELAQHGQISETNVYNWFQNRRARSKRKQSALLPNSGESEVETEIEPFKEKKTKPEDNQPDEDATPVSDHMYLHSPDIGIDQLVGKMESPGSCIPYWQLEQYDLFG

>PotriWOX13b

SRQRWTPKPAQLQILEQIFEQCNATPGRQKIKDITRELAQHGQISETNVYNWFQNRRARSKRKQSAVVPNNGESEMETDIESLKEKKTRAEDSQPDENTTPMADHMYFNSPDIGFDQLMGKIESPGSCIPYWQMEQYDLFG

>AtWOX5

KCGRWNPTVEQLKILTDLFRAGLRTPTTDQIQKISTELSFYGKIESKNVFYWFQNHKARERQKRRKISIDFDHHHHQPSTRDVFEISEEDCQEEEKVIETLQLFPVNSFEDSNSKVDKMRARGNNQYREYIRETTTTSFSPYSSCGAEMEHPPPLDLRLSFL

>AtWOX7

KCGRWNPTVEQVKLLTDLFKAGLRTPSTDQIQKISMELSFYGKIESKNVFYWFQNHKARERQKCRKISTVKFDHRQDTDLSKPRRDNVRRHQLPAKG

>PotriWOX5a

KCGRWNPTTEQVKLLTDLFRSGLRTPSTDEIQNISTQLSFYGKIESKNVFYWFQNHKARERQKRRRVSVDEKDAMIHFTEINHVNEPERVIETLQLFPLNSFDEAGPEKFRFQANECNEAAAAFSYKFGTEMDHPHLDLRLSFV

>PotriWOX5b

KCGRWNPTIEQGKLLTDLFRSGVRTPSTDEIQNISTRLSFYGKIESKNVFYWFQNHKARERQKRRRVSVDEKDVMIRRDDKFSSARYFTEIGQVNEREQVIETLQLFPLKSFDEVESEKFRLQANECNEAAAAFSYKFGTEMDRPQLDLRLSFL

>PheWOX5b

KCGRWNPTAEQVKVLTELFRAGLRTPSTEQIQRISTHLSAFGKVESKNVFYWFQNHKARERHHHKKRRRGASSPDSGSNEEDGRPAAREAEADLVLQPPESKREARSYNHHRLMTCYVRDVVEQEAMLERPTREVETLELFPLKAYDLEADKVLYVRGGGGGEQCRESSFFDVVAGRDPPLELRLCSFGL

>PheWOX5c

KCGRWNPTVEQVKVLTELFRAGLRTPSTEQIQRISTHLGAFGKVESKNVFYWFQNHKARERHHHKKRRRGASSPDSGSNEEDGRAAASEAEADLVLQPPESKREVRSYSHHRLMTCYVRDVVEQEAMSERPTREVETLELFPLKAYDLEADKVRYVRGGGGGGEQCREISFFDVAAVRDPPLELRLCSFGL

>OsWOX5

KCGRWNPTAEQVKVLTELFRAGLRTPSTEQIQRISTHLSAFGKVESKNVFYWFQNHKARERHHHKKRRRGASSPDSGSNDDDGRAAAHEGDADLVLQPPESKREARSYGHHHRLMTCYVRDVVETEAMWERPTREVETLELFPLKSYDLEVDKVRYVRGGGGEQCREISFFDVAAGRDPPLELRLCSFGL

>GRMZMWOX5a

KCGRWNPTAEQVKVLTELFRAGLRTPSTEQIQRISTHLSAFGKVESKNVFYWFQNHKARERHHHKKRRRGASSSSPDSGSGRGSNNEEDGRGAASQSHDADADADLVLQPPESKREARSYGHHHRLVTCYVRDVVEQQEASPSWERPTREVETLELFPLKSYGDLEAAEKVRSYVRGSGATSEQCRELSFFDVVSAGRDPPLELRLCSFGP

>GRMZMWOX5b

KCGRWNPTPEQVKVLTELFRAGLRTPSTDQIQRISAHLGAFGKVESKNVFYWFQNHKARERHHHKKKKRRRRGAAMASSSSPDSGSGSGSSEEDGRVAAAADADLVLQPSPGRSKSKREARRSYGGGHHRRLVTCKQLAHTHRYVRDVVEQQEEDTWERPTREAVETLELFPLKSYVDLEAEKVRRYVVRAGASEQCRDFSFFDVSGGRDPPLLELRLCSFGP

>BradiWOX5

KCGRWNPTAEQVKVLTELFRAGLRTPSTEQIQRISTHLGAFGKVESKNVFYWFQNHKARERHHHKKRRRVASSSSDNSSASNNNDEAADHGRSSAREDLLLQPPESKREARSYNHHRRPIMTYVGYVRDEVEQEVVMWERPTREVETLELFPLKAAYDLEAADRLRYVRGAGEQQCREISFFDVANGRDPPLELRLCSFDI

>PheWOX5a

KCRRWSPTAEQVKVLTALFRSGLRTPSTEQIRRISDHLGAFGKVESKNVFYWFQNHKARERHHHHHRKRGRRGRGACWTVQNSGHEGVGGEARTYSHRLMTCDLRGAVLEQEPDMWERPMRELETLELFPLNSYTVDVEEAPYTTNGSGVQCRDALLLGAPGRDDPPLALRLSCGGL

>PotriWOX4-1

GGTRWNPTQEQIGILEMLYRGGMRTPNGQQIEDITAQLSRYGKIEGKNVFYWFQNHKARERQKQKRNSLGLSHSPRTPSPITIISLDTRGEVEKDEDSPYKRKCRSWSFECFELEESRSCKEEGDRTLELFPLHPEGR

>PotriWOX4-2

GGTRWNPTQEQIGILEMLYRGGMRTPNGQQIEDITAQLSRYGKIEGKNVFYWFQNHKARERQKQKRNSLGLSHSPRTPSPVTIISLDTRGEVEGEEDSPYKRKCRSWTFECLELEDSRSCREKGDRTLELFPLHPEGR

>AtWOX4

GGTRWNPTQEQIGILEMLYKGGMRTPNAQQIEHITLQLGKYGKIEGKNVFYWFQNHKARERQKQKRNNLISLSCQSSFTTTGVFNPSVTMKTRTSSSLDIMREPMVEKEELVEENEYKRTCRSWGFENLEIENRRNKNSSTMATTFNKIIDNVTLELFPLHPEGR

>PheWOX4a

TTTRWNPSPEQIKVLEMLYCGGMRTPNALQIERITEELSKYGRIEGKNVFYWFQNHKARERQKQKRAALLTLSTLDSSSLPETKDGEEKKEMCDDMTSCKRPCKTRGDGHGDAATEVADCTDDVTLELFPLHPQGKA

>PheWOX4b

TTTRWNPSPEQIKVLEMLYCGGMRTPNALQIERITEELSKYGRIEGKNVFYWFQNHKARERQKQKRAALLTLSTLDSSSLPETKDGEEKKEMCDDMTSCKRPCKTRGDGHGDAATEVADCTDDVTLELFPLHPQGKA

>PheWOX4c

TTRWNPSPEQIKVLEMLYRGGMRTPNALQIERITEELGKYGRIEGKNVFYWFQNHKARERQKQKRAALLTLSTLDSSALPQTKDGAEKMEVYCDDVASCKRRCKTWGDGHGDAATEVADCTDDVTLELFPLRPRGKA

>OsWOX4

TTRWNPSAEQIKVLEMLYRGGMRTPNSVQIERITEELGKYGRIEGKNVFYWFQNHKARERQKQKRAALLTLSTLDPSLLPATANETKEAPEKKEKDVEDGLASCKRRCKAWGDGAGDGDAVVATEAAGGCTDEVTLELFPLHPQGKA

>BradiWOX4

TTRWNPSTEQIKVLEALYRGGMRTPNAAQIERITEELGRHGRIEGKNVFYWFQNHKARERQKQKRAALLTLATAGTGLDDSSDSPPPPPETTTTTKDGAEKMEAAACADVDVTTSCKRRCKAWGDGGHGGAAETEGGGAADDVTLELFPLRPQGKA

>AtWOX1

VSSRWNPTPDQLRVLEELYRQGTRTPSADHIQQITAQLRRYGKIEGKNVFYWFQNHKARERQKRRRQMETGHEETVLSTASLVSNHGFDKKDPPGYKVEQVKNWICSVGCDTQPEKPSRDYHLEEPANIRVEHNARCGGDERRSFLGINTTWQMMQLPPSFYSSSHHHHQRNLILNSPTVSSNMSNSNNAVSASKDTVTVSPVFLRTREATNTETCHRNGDDNKDQEQHEDCSNGELDHQEQTLELFPLRKEGFCSDGEKDKNISGIHCFYEFLPLKN

>PotriWOX1a

MSSRWNPTPEQLRTLEELYRRGTRTPSTDQIQDITAQLRRYGRIEGKNVFYWFQNHKARERQKRRRQMESDSLDDHQQNGHGVEMFERKEPGASMTGYEGEQTRNWAPSTNCSTLSEESVSISKATKAAMAEYYRPDGWIEFDEGEIMQHRRNLIERNATWEMMPFSCPSPTHLLNTISSATATTIATTSASTQGAATVRTMDPTKLMNAHDLNIFIAPYIENGYHGARINHFNNSVINEGGEYCRDGNDESQTLQLFPIRSGGNGNNIERINERETEVSVSATETLNANDFSPCQFFEFLPLRI

>PotriWOX1b

MSSRWNPTPEQLRTLEDLYRRGTRTPSTDQIQDITAQLRRYGRIEGKNVFYWFQNHKARERQKRRRQMESDSFDGHLQNGHGIEIFERKESEASRTGYEGEQTKNWAPSTNCSTLSEESVSISRATKGAMAEYCRPDGWMQLDEGELQHRRNFIERNATWEMMQLSCPSPTHQRNTISSTSSTTTMSKQGAAAAKLIKAHDLNVFIAPYRENGHHGALINQFNSSVINDGDESRGGTGESQTLQLFPLRSGGDGNNNIESINERESEVSVSAAEALNANNFAPCQFFEFLPLKH

>PotriWOX6

RSSRWNPTAEQLLALEEKYSCGVRTPTTNQIQQITSELRRFGKIEGKNVFYWFQNHKARERQKHRQVQQKHNNTDHESSNKMKESGPRRTVLGVDQTNNLAPHSKCSTDHVEGPVSVNGAAIAESGTHGWSEFEERELQQMKSISLDMHAMWQTMDLSSSTPVHRLTSTMTTTASKFSSLEEHSSLLRPTKTATHANHDGEIREVQTLQLFPLCSDDGNGANGTNNDRNVPIRTINTTFTPSQFFEFLPLKN

>AtWOX6

ATLRWNPTPEQITTLEELYRSGTRTPTTEQIQQIASKLRKYGRIEGKNVFYWFQNHKARERLKRRRREGGAIIKPHKDVKDSSSGGHRVDQTKLCPSFPHTNRPQPQHELDPASYNKDNNANNEDHGTTEESDQRASEVGKYATWRNLVTWSITQQPEEINIDENVNGEEEETRDNRTLNLFPVREYQEKTGRLIEKTKACNYCYYYEFMPLKN

>PotriWOX2a

GNSRWNPTKEQISMLESFYSQGIRTPSTEMIEQITSRLKAYGHIEGKNVFYWFQNHKARQRQKQKQESMAYINNYLHKVHQPVFAPPCANVVCSPYFPQQSEVMGFCQQHPKMLLPSNFKMRPRSEARTYAFNGYEPAAPYGYHNRITMNKGERTLVTINHKSSSDQATLPLFPLHPTGTLEGATSICPVGSTDPAENSTNTPSSSEITTGIEEHSGDCKPFFDFFYGKDS

>PotriWOX2b

VNSRWSPTKEQISMLESFYSQGIRTPSTEMIEQIASRLKAYGHIEGKNVFYWFQNHKARQRQKQKQENMAYINKYLHKAHQPVFAPPCRNVVNSPCYLPKSDIMGLCQQHQNMLLPGNFKRRSRSETISYAFKGYDQEAVLREYHNHITKNKFERSPVTIDKSSSDQETLPLFPLHPTGILEGASPIFSHGSTSAENSINTPISSEITHGIGEHSADHKPFFDFFSEKDPFESSH

>AtWOX2

SSSRWNPTKDQITLLENLYKEGIRTPSADQIQQITGRLRAYGHIEGKNVFYWFQNHKARQRQKQKQERMAYFNRLLHKTSRFFYPPPCSNVGCVSPYYLQQASDHHMNQHGSVYTNDLLHRNNVMIPSGGYEKRTVTQHQKQLSDIRTTAATRMPISPSSLRFDRFALRDNCYAGEDINVNSSGRKTLPLFPLQPLNASNADGMGSSSFALGSDSPVDCSSDGAGREQPFIDFFSGGSTSTRFDSNGNGL

>GRMZMWOX2

ANARWNPTKEQVAVLEGLYEHGLRTPSAEQIQQITGRLREHGAIEGKNVFYWFQNHKARQRQRQKQDSFAYFSRLLRRPPPLPVLSMPPAPPYHHARVPAPPAIPMPMAPPPPAACNDNGGARVIYRNPFYVAAPQAPPANAAYYYPQPQQQQQQQVTVMYQYPRMEVAGQDKMMTRAAAHQQQQHNGAGQQPGRAGHPSRETLQLFPLQPTFVLRHDKGRAANGSNNDSLTSTSTATATATATATASASISEDSDGLESGSSGKGVEEAPALPFYDFFGLQSSGGR

>GRMZMWOX2-like

ARWNPTKEQVAVLEGLYEHGLRSPSAEQIQQIADRLREHGHGHGAIEGKSVFYWFQNHRARLRQQRQKQESFAYFTRLLRRPPPLPVLSMPPAPPYRHGRVPSPATITMPAPPPPTPAACYNSGGSR

>OsWOX2

ANARWTPTKEQIAVLEGLYRQGLRTPTAEQIQQITARLREHGHIEGKNVFYWFQNHKARQRQKQKQQSFDYFSKLFRRPPPLPVLHRPLARPFPLAMAPTAMPPPPPPPATTTTAACNAGGVMFRTPSFMPVATNNASYYPQQQTPLLYPGMEVCPHDKSTAQPPATTTMYLQAPPSSAHLAAAAGRGAAEAEGHGRRGGGAGGRETLQLFPLQPTFVLPDHKPLRAGSACAAVSPTTPSASASFSWESESSDSPSSEAPPFYDFFGVHSGGR

>PheWOX2

WTPTKEQIAVLEGLYGQGLRTPTAEQIQQITARLREHGHIEGKNVFYWFQNHKARQRQKQKQQSFDYFSKLLRRPPPLPVLHRAPEPPFPVPLPAPQITMPPPVSAHPACNNGVMYSPPCYMPAPQAAANAAYYPHPQMPGLYRVQAPTTMYLQPAPNNAGTPLHLFPLQPTFLLSDKARRTGSASLTPTPSASASFSGESESSERPNGEALPFYNFFGVHSGGC

>BradiWOX2

ANARWTPTQEQRELLEGLYRQGLHTPSAEQIQGIAARLRQHGPVEGKNVFYWFQNYKARQRQRQRLQGLAYFDREFRRPMPIPVLHRFPSPPATAPVPLLPAACNRSEANMYRQPSFFPQTPQAAANATAAHYLQTQPPLLYPGFGNAPALSRYQQPAPNSAGGSGTQQLRGMRFPSASGAANASSGSGTRDRETLQLFPLQPTCWQMREKKKKNCSSTGSGSPSPMTPSSSGSASSSFSLEPESPEVPFYDFFGLQSGGRAD

>AtWUS

TSTRWTPTTEQIKILKELYYNNAIRSPTADQIQKITARLRQFGKIEGKNVFYWFQNHKARERQKKRFNGTNMTTPSSSPNSVMMAANDHYHPLLHHHHGVPMQRPANSVNVKLNQDHHLYHHNKPYPSFNNGNLNHASSGTECGVVNASNGYMSSHVYGSMEQDCSMNYNNVGGGWANMDHHYSSAPYNFFDRAKPLFGLEGHQEEEECGGDAYLEHRRTLPLFPMHGEDHINGGSGAIWKYGQSEVRPCASLELRLN

>GRMZM2G010929-P02

GGAGGGSGSVVASAVCRPSGSRWTPTLEQIRMLKELYYGCDIRSPSSEQIQRITAMLRQHGKIEGKNVFYWFQNHKARERQKRRLTGSTSWPRGGRGARLERGRDRDGRGVVRGDGGRGRDSARRGEGRGDGAQGGDDAGGAQIAWRGGANRVRGGRR

>PotriWUS1

TSTRWTPTTDQIRILKELYYIKGVRSPNGAEIQQISARLRKYGKIEGKNVFYWFQNHKARERQKKRFTNDVPTQQRTTLKPEDYYSYKYSGSNNNPGFSSASSSSNTGAVTVGQADNYGYGSVTMQEKKNWDCSVPAGGESMNNINYGSRGGIYPYSSSYTVFDQDQEAAEKIETLPLFPMHGEDISTSFNINNVNPDFYYSSWYGSDDYGNATTSRTSLELSLYSYNGQQQDY

>PotriWUS2

TSTRWNPTTDQIRILKELYYIKGVRSPNGAEIQQISARLRKYGKIEGKNVFYWFQNHKARERQKKRLTNEVPMQQRTAWKPEDYYSYKYSNSNNNPGFSSASSSANTGVVTVGQTDSHGYGSVTMQEKNSWDCSAPAGGSNGAGSGSMSNINYGSGVDINSHSSSYAVFGQEQEAAAKIETLPLFPMLGEDISSSFNINNINPDFYYSSGCGYGDYGNDTSSRTSLDLSLYSYNGQPQDY

>GRMZMWUS1

SGSRWTPTPEQIRILKELYYGCGIRSPNSEQIQRITAMLRQHGKIEGKNVFYWFQNHKARERQKRRLTNLDVNVPVAADDSAHRLGVLSLSPSSGCSGAAPPSPTLGFYAGGNGSAVMLDTSSDWGSAAAMATEACFMQDYMGVMGGASPWACSSSSSEDPMAALALAPKVTRAPETLPLFPTGGGGDDRQPPRPRQSVPAGEAIRGGSSSSSYLPFWGAAPTPTGSATSVAIQQQHQLMQMQEQYSFYSNAQLLPGTGSQDAAATSLELSLSSWCSPYPAGTM

>GRMZMWUS2

SGSRWTPTPEQIRMLKELYYGCGIRSPSSEQIQRITAMLRQHGKIEGKNVFYWFQNHKARERQKRRLTSLDVNVPAAGAADATTSQLGVLSLSSPPSGAAPPSPTLGFYAAGNGGGSAGLLDTSSDWGSSGAAMATETCFLQDYMGVTDTGSSSQWPCFSSSDTIMAAAAAAARVATTRAPETLPLFPTCGDDDDDDSQPPPRPRHAVPVPAGETIRGGGGSSSSYLPFWGAGAASTTAGATSSVAIQQQHQLQEQYSFYSNSTQLAGTGSQDVSASAAALELSLSSWCSPYPAAGSM

>PheWUS

SGTRWTPTSEQVRILRELYYNCGIRSPNSEQIQRITAMLRQYGRIEGKNVFYWFQNHKARERQKKRLTTLDVNTSSVAAGAADVNHLGVLSLSPSGAAAPSSPGLYSGNGASAVQQLEASTNCWDSSTAMATKRSFLQVISRATSTDTLQYRSCTPIEFP

>PheWUS-like

MLRQYGRIEGKNVFYWFQNHKARERQKKRLTTLDVNTSSLAAADVNHLGALSLSPSGATAPSSPGLYAGNGASAVQQLEASTNCWDSTAMTTERSFLQQEYNLYHCQFFISAFQNVIH

>OsWUS

SGTRWTPTTEQIKILRELYYSCGIRSPNSEQIQRIAAMLRQYGRIEGKNVFYWFQNHKARERQKKRLTTLDVTTTTAAAADADASHLAVLSLSPTAAGATAPSFPGFYVGNGGAVQTDQANVVNWDCTAMAAEKTFLQDYMGVSGVGCAAGAAPTPWAMTTTTREPETLPLFPVVFVGGDGAHRHAVHGGFPSNFQRWGSAAATSNTITVQQHLQQHNFYSSSSSQLHSQDGPAAGTSLELTLSSYYCSCSPYPAGSM

>BradiWUS

SGTRWTPTAEQVRILRELYYGLGIRSPNAEQIQRIAGRLRQYGRIEGKNVFYWFQNHKARERHKKRLTTIDVSPNNNDSNNNCSNNASILSLSPSSGAAAGLYGAGSNGGGGSAHLQMDANASATCWDVGSNAAMANDRSFMQLLQEQDYMGVRTSTAAMAPTPWPACFPAFSPYQPPPAREPETLPLFPTGGGSSGGHQEIVNGVHGGSYQLLQSNSQQLCWGQQHHHHHQLLLQEQQNYQYSSYSSNNQLMMPTQDAAAAASLELTLSSHYPAGSSM

>BradiWOX3a

TRWCPTPEQLMILEEMYRSGVRTPNAAEIQQITAHLAYYGRIEGKNVFYWFQNHKARERQRLRRRLCARHQQQPPASSPSPVTPPPPNASAGAVNVMHPAVMQLHHHHHHHPYATTTTCSFMPPQGYSHQQQQDAGGAVPVSGLEFPAAGGGKAHQQQQEWTTQQQQQMMMMENSNNINAGGSSGAGGMTPPPWPCCRPLRTLELFPTKSTGGGLRDECSSSKSSSCSTSTN

>OsWOX3a

TRWCPTPEQLMILEEMYRSGVRTPNAAEIQQITAHLAYYGRIEGKNVFYWFQNHKARERQRLRRRLCARHQQQPSPPSSTVPPAPTAAAAGAVVQVHPAVMQLHHHHHHHHPYAAAAAAQSHHLQQQQQQQAEWPAAVDYCSTASASASATAADMAIPPCCRPLKTLELFPTKSTSGGLKEDCCSSSKSSSCSTSTN

>GRMZMWOX3a

TRWCPTPEQLMILEEMYRSGVRTPNAAEIQQITAHLAYYGRIEGKNVFYWFQNHKARERQRLRRRLCARHQQQYAQQQATAAAPASSPNSSATVPSLAAGGSSAGVHPAVMQLHHHQHPYATNFMPHQLGYMGQQVATVPPVLNPAAAGMVDLAAARAGGGNKATAAGSGAYGGGAGLYNSCSSNQLEEWEATDAMEHCDASCGAASGSSDEGGALQLPPCCRRPLKTLDLFPTKSTGLKDECSSSKSSSCSTSTN

>PheWOX3a

TRWCPTPEQLMILEEMYRSGVRTPNAAEIQQITAHLAYYGRIEGKNVFYCGGSGGAAEGSVHPAVMQLHHHHPYATTFMPQGGYFQQDAAAVSGGTEFAGCKTGGDPIGGGGGGYSNNNQQQQDWAAQMMMDCGAGGSSDTIPPCCRPLKTLELFPTESTGLKDECSSSKSSSCSTSTN

>PheWOX3a-like1

TRWCPTPEQLMILEEMYRSGVRTPNAAEIQQITAHLAYYGRIEGKNLLLLLTLPIMLLPAPVAVVGTAALLQAVCIPL

>PheWOX3a-like2

TRWCPTPEQLMILEEMYRSGVRTPNAAEIQQITAHLAYYGRIEGKNVFYWFQNHKARERQRLRRRLCARHQQQYAQQQAPSPASALAAAPPPNAANNSAAAGGSSTGSGGGAAAGGMHPAVMQLHHHHPYATSFMPQGGYFQQDATAAALPVVSAGMEFAACKAGDPNGGTGGGAYGGGIILNYGQHKIIGYS

>GRMZMWOX3b

STRWCPTPEQLMVLEETYRGGLRTPNASQIQQITAHLACYGRIEGKNVFYWFQNHKARDRQKLRRMLFMSQSHHLLSCAQYYAAVLAPRHGHQLLLSPSSTSPTPPAAAAAAAYGYYYSATAFAEPASSGGGLVPAEALGRPEYSSLDNFGVALDDVVVSSASAAVEMTPPGFEVVVPPPPAAAFCRPLKTLDLFPCGLKEEQHDVA

>GRMZMWOX3-like

MILEDMYRGGLRTPNASQIQQITAHLACYGRIEGKNVFYWFQNHKARDRQKMRRRLCMSHHLLSCAQYYAAAAHHGHAAFLAAPPPYGHQLLSPSTTSPTPAAAAAAAAAAYGYYYPATAAFAAPASRCAGNATPPSPTTQLFHYQGGGGLVPTEALGRPEYSLGKLDNFGVALDDVVVSSTSGAVVDTMGAPPVAGFEVAPPLPAAFSCRPLKTLDLFPGGLEEEQHDVA

>OsWOX3b

STTRWCPTPEQLMMLEEMYRGGLRTPNAAQIQQITAHLSTYGRIEGKNVFYWFQNHKARDRQKLRRRLCISHHLLSCAHYYHHHLAAAAAVVPPPQLLPPLHPSSSSSSCGGGLIDHANSLLSPTSATTPTSAAAAAAAAAYTTSYYYPFTAAAAPPPPRTSPAASPLFHYNQGGGGVVLPAAEAIGRSSSSSDYSLGKLVDNFGVALEETFPAQPQQPATTMAMTAVVDTTAVAAAAGGFCRPLKTLDLFPGGLKEEQHDVV

>PheWOX3b1

STRWCPTPEQLMILEDMYRGGLRTPNSSQIQQIAAHLAFYGRIEGKNVFYWFQNHKARDRQKLRRRLCMSHHFLSCAHYYAAAAAAGQQQQQLLGTAAIAGAPPPLCTPPPAPHYSCLDQQAAAAHLTTAPAAAYTSYYYPFGAAPASRCTAGAGATPSPPNPLLQYQGGGGRGVLPEAIGRPEYSLGKLGNFGVVEDTSGCPASTVVDIPPPSGYEAAMEAAGEAMSAPSTAFCRPLKTLDLFPGGLKEEQRDVV

>PheWOX3b2

STRWCPTPEQLMILEEMYRGGLRTPNASQIQQITAHLAFYGRIEGKNVFYWFQNHKARDRQKLRRRLCMSHHLLSCAQYYAAAAAGHHHHQLLGATAIAGAPPHLTPPAPHYSCLDQQAAAGHLTTAPAAAYGSYYYPLGAAAAPASRCTGATPSPPNPLFLYQGGGGGVVLPEYSLGKLDTSGCPASTVVDTPPPLGYEAAMEAAGDAMSVPSTAFCRPLKTLDLFPGGLKEEQRDVV

>BradiWOX3b

TTRWCPTAEQLMVLEEMYRGGLRTPNASQIQQITAHLAHYGRIEGKNVFYWFQNHKARDRQKLRRRLCMTHHLLSCAAAAAAVQQAQHHHYYAAAAAAGQFLGGPAGGGAGHPMAQQQQQQYYSAASAMACPAGGFDQHHYQLLPASPMAAACYPAQLQPSSRCAGGVSPVPAPAQPNQFLQYQQQGGGSPEFSLGRLGSFGVVVDQEEADTCRSGYDYQQQLAAARMEAAETTSFCRPPLKTLELFPGASVKDEQLA

>AtWOX3

TRWCPTPEQLMILEEMYRSGIRTPNAVQIQQITAHLAFYGRIEGKNVFYWFQNHKARDRQKLRKKLAKQLHQQQHQLQLQLQQIKPKPISSMISQPVNKNIIDHHNPYHHHHHNHHHNHHRPYDHMSFDCCSHPSPMCLPHQGTGVGEAPSKVMNEYYCTKSGAEEILMQKSITGPNSSYGRDWMMMMDMGPRPSYPSSSSSPISCCNMMMSSPKIPLKTLELFPISSINSKQDSTKL

>AtWOX11

VRSRWSPKPEQILILESIFHSGMVNPPKEETVRIRKMLEKFGAVGDANVFYWFQNRRSRSRRRQRQLQAAAAAADATTNTCDQTMMVSNSLPHHSGSDLGFGGCSTSSNYLFASSSSSYGGGCDNQSNSGMENLLTMSGQMSYHEATHHHYQNHSSNVTSILCPSDQNSNFQYQQGAITVFINGVPTEVTRGGIDMKATFGEDLVLVHSSGVPLPTDEFGFLMHSLQHGEAYFLVPRQT

>AtWOX12

VRARWSPKPEQILILESIFNSGTVNPPKDETVRIRKMLEKFGAVGDANVFYWFQNRRSRSRRRHRQLLAATTAAATSIGAEDHQHMTAMSMHQYPCSNNEIDLGFGSCSNLSANYFLNGSSSSQIPSFFLGLSSSSGGCENNNGMENLFKMYGHESDHNHQQQHHSSNAASVLNPSDQNSNSQYEQEGFMTVFINGVPMEVTKGAIDMKTMFGDDSVLLHSSGLPLPTDEFGFLMHSLQHGQTYFLVPRQT

>PotriWOX11a

VRSRWTPKPEQILILESIFNSGMVNPPKDETVRIRKLLEKFGSVGDANVFYWFQNRRSRSRRRQRQMQASLVAGEQTNNQQAQASGGAIQYKGCNTSIGFANSPSFVQSPSSYLVGSSSSYGVVDEDHGGESLYSFSNQMAFQEVEQTSGVTSILYPSETSNLHYQTAGFITVFINGIPTEVPRGPLDIKAMFGQDVVLVHSSGVPVPTNEFGFLMQSLHHGESYFLVIISGA

>PotriWOX11b

VRSRWTPKPEQILILESIFNSGMVNPPKNETVRIRKLLEKFGSVGDANVFYWFQNRRSRSRRRQRQMQASGGTSNGFANSPSSYLVGASSSCGVVGEDHGVESLFSFSNQMGFQEFEQTSGVTSIVCPSETSSLHYQTAGFITIFINGVPTEVPRVPLDVKAMFGQDVMLVHSSGVPVPTNEFGFLVQILHHGESYFLVNISAV

>GRMZMWOX11a

VRSRWTPKPEQILILESIFNSGMVNPPKDETVRIRKLLERFGAVGDANVFYWFQNRRSRSRRRQRQMQAAAAAAATAAAASSAANSSPGASATVGLPSGALQYPLVMSGSGTACQYEQQASSSSSSGSTGGSSLGLFALGAGVPSTGGGFFQESCGASSPLATGLMMGDVDRSGGSDDLFAISRQMGFAAASPVASASVAPPSTTAHHQQYYSCQSPTGTITVFINGVPMEVPSGPIDMRAIFGQDVLLVHSTGALLPVDDYGILTQSLQTGESYFLVARPT

>GRMZMWOX11b

ARSRWTPKPEQILILESIFNSGMVNPPKDETVRIRKLLERFGAVGDANVFYWFQNRRSRSRRRQRQMQAAAAAAAAAAASSASNNSSPAASATVGLPSGALQYPLAMGGTAACQYEQQASSSSSSGSTGGSSLGLFALGAAGVPGTGGGGYFQASCGASSPLATGLMGDVDSSGGSDDLFAISRQMGFAAASTVASASVAPSSNAHHQQYYSCESPAATITVFINGVPMEVPRGPIDLRAMFGQDVMLVHSTGVILPVNDYGILTQTLQLGESYFLVARPT

>OsWOX11b

VRSRWTPKPEQILILESIFNSGMVNPPKDETVRIRKLLERFGAVGDANVFYWFQNRRSRSRRRQRQMQAAAAAAAAAASSSSPSANTSPAAASAATVQVGLPPGAVVHTMAMGGSACQYEQQASSSSSSGSTGGSSLGLFAHGAGASGAGGYLQASCGASASASSALAPGLMGDVVDSGGSDDLFAISRQMGFVGSPRCSPASSPATPSSAATAAQQQFYSCQLPAATITVFINGVPMEMPRGPIDLRAMFGQDVMLVHSTGALLPVNDYGILMQSLQIGESYFLVARPP

>PheWOX11-like1

MVNPPKEETVRIRKLLERFGAVGDANVFYWFQNRRSRSRRRQRQMQAAAAAAVAGASSSAANSSPAASATVGLPSVAVHPMAMGRSACQYEQQASSSSSSGSTGGSSLGLFAHGAGVSGAVDGGYLQASCGASSALASGLMGDVDSGGSDDLFAISRQMGFVENPRGSSSSAPSIAAQRQYYSGQLPAATITVFINGVPMEVPRGQIDLRAMFGQDVMLVHSTGALLPVNGYGILMQSLQMGESYFLVTRTT

>BradiWOX11b

VRSRWTPKPEQILILESIFNSGMVNPPKDETVRIRKLLERFGAVGDANVFYWFQNRRSRSRRRQRQMQAAAAAAAAANNNNTSSAAAASATIGGQLPSAMAIVGGSACQYEQQASSSSSSGSTGGSSSLGLFAHGAAGVSSSGPGAGVGYQQLLQQQQAASCGASLSALANSGLMVGDVGDSGGGDDLFAISRQMGFVDHSPVGSSNSSAAPSTAVQQQQQYFSCQLPTATITVFINGVPMEVPRGPIDLRAMFGQDVVLVHSTGALLPVNDYGILIQSLQMGESYFLVARQT

>PheWOX11

VRSRWTPKPEQILILESIFNSGMVNPPKDETVRIRKLLERFGAVGDANVFYWFQNRRSRSRRRQRQLQAQAAAAAASSGSPPASGAGHAPGQAGSASSLGLFAHGPAYSSSVSSSWTSSPSSAGLMGDVDCGGGDDLFAISRQMGYMDGGGSGSSSSAAAAQQQQQLYYSCQPATITVFINGVATEVPRGPIDLRSMFGQDVMLVHSTGGLLPVNEYGILMQSLQMGESYFLVTRST

>PheWOX11-like2

MSIGTVNPPKDETVRIRKLLERFGAVGDANVFYWFQNRRSRSRRRQRQLQAQAQAAAAAAPGSPPASGAGHASGHVSSASSLELFAHGAAYSSSASSSWPSSPPSAGLMGDVDCGGGDDLFAISRQMGYVDGGGSGSSSSAAAAQQQSYYSCQPATITVFINGVATEVPRGPIDLRSMFGQDVMLVHSTGGLLPVNENGILMQSLQMGESYFLVTRPA

>OsWOX11a

VRSRWTPKPEQILILESIFNSGMVNPPKDETVRIRKLLERFGAVGDANVFYWFQNRRSRSRRRQRQLQAQAQAAAAAASSGSPPTASSGGLAPGHAGSPASSLGMFAHGAAGYSSSSSSSWPSSPPSVGMMMGDVDYGGGGDDLFAISRQMGYMDGGGGSSSSAAAGQHQQQQLYYSCQPATMTVFINGVATEVPRGPIDLRSMFGQDVMLVHSTGALLPANEYGILLHSLQMGESYFLVTRSS

>BradiWOX11a

VRSRWTPKPEQILILESIFNSGMVNPPKDETVRIRKLLQRFGPVADANVFYWFQNRRSRSRRRQRQLQQQAQQLAQAHQAPAALSQSAPVAAQYGGGGYSSSSSSSSTWPPSSPPSAGIMMDGGGGDDLFAISRQMGYGSGSSSSAPAAMAHEQSQMYYGYSYQQQAAGGMNNSMIQVYINGVATEVPRGPVDVRAMFGDDAVLVHAAGGMLPVDDYGVLLHSLQMGESYFLVSRSA

>PheWOX12b

RSRWAPKPEQILILESIFNSGKVNPAKDDTARIRRLLERFGAVRDANACGGAQLPATAGGHDVSTSAFIMHGQLCAGMSTAAAPVVPSAAASSHFWAGDVVDGGDDLFAISRQMGLMARGGDHHYSCMATDASQLSYQPTGTIQVFINGAAYEVPSAGALDLAGTFGRDAMLVHSSGEILPVNEHGVLVKSLQMGECYYLVSRSI

>PheWOX12c

RSRWAPKPEQILILESIFNSGMVNPAKDDTARIRRLLERFGAVRDANACGGAQLPATAGGHDVSTSAFIMHGQLCAGMSTAAAPVVPSAAASSHFWAGDVVDGGDDLFAISRQMGLMARGGDHHYSYMATDASHLSCQPTGTTIQVFINGAAYEVPSAGALDLAGTFGRDAMLVHSSGEILPVNEHGVLVKSLQMGDCYYLVSRSI

>PheWOX12a

RSRWAPKPEQILILESIFNSGMVNPAKDETTRIRRLLEGFGAVRDANVFYWFQNRRSRARRRARQLQQAYGGAQLPATAAGHHDINTSPFIMHGQLCSGVPTAAPVPSAAAVTAASHFWTDDDIDGGDDLFAISRQMGLMARGGDHHYSYTATDSSQLTYQPTGTIQVFINGTAYEVPSAGALDLAGTFGRDAMLVHSSGEVLPVNEHGVLMKSLQMGECYYLVARSI

>OsWOX12

RSRWAPKPEQILILESIFNSGMVNPAKDETARIRRLLERFGAVRDANVFYWFQNRRSRSRRRARQLQQACGAALHQLPSAAAAAGAGGGGDYYHHHHQPSSSPFLMHGGGGGGVVTSTTAAPAVAASGHFLADEVDGGGDDDLFAISRQMGLMARHGGGDHHYSSYADSDATQLSYQPTGTIQVFINGVAYDVPSGGALDMAGTFGRDAMLVHSSGEVLPVDEHGVLINSLQMGECYYLVSKSI

>GRMZMWOX12

ARSRWAPKPEQILILESIFNSGMVNPAKDETARIRRLLERFGAVRDANVFYWFQNRRSRSRRRARQLQQQQAGSGPQHPTAAAAAGHVHGANANDGVSLYAMHGHAGQQQQARAAGLPAVAPVVLPPAAVASSPRFFGDEIDTDGGDDLFAISRQMGLMSRGGEQHGCGYVANNDASQSHLLSYQPTGTATTAAAAYIQVSINGVVYEVPGAGALLDLAGTFGRDAMLVHSSGEILPVNENGVLMKSLQMGECYYLVSKST

>BradiWOX12

RSRWAPKPEQILILESIFNSGMVNPAKDETARIRRLLERFGAVRDANVFYWFQNRRSRSRRRARQLQHQAQLHQLPSAPHGIMHYGQLSCGGGGVMSPAAAPVASAPAPTAGPHLLADVVGAGDDDLFAIPRQMGLMDHRYTETSQLTYQQPGTIQVFINGAAYDVPSAGPLDLAGTFGHDVMLVHSSGEILPVNEHGVLMKNLQMGECYYMVSRSI

>PH02Gene09575.t2

GGWGAGEPVRSRWTPKQEQVLILESIFNSGMVNPPKEVTVRIHKLLKRFGTVGDANVFYWRGGLAARSQRRRGKRRIMRKTNSQSHLCLTSQPRKR

>PotriWOX9a

PKPRWNPKPEQIRILEAIFNSGMVNPPRDEIRKIRAQLQEYGQVGDANVFYWFQNRKSRSKHKLRNLQNSKQQITPSTTKPVTASLTAPSSLSSSSEKSSPKVSKRTLSLSSPPFIDASNSPNSSVSQTYFQAQNEFVSEPFFFPVQQTGGETVAFTQGFCFSELSNVVHVQDHTVGPCPSLLLSEITNSSASKKANHEERNLKMQPQLSYTATSPVTHSIDLAPPLPLSANTSTVSIQSTISQIQGLGVSGGNERSTVFINDVAFEVAMGPFNVREAFGDDILLIHSSGQPVLTNEWGITLDSLQHGALYYLVPLSISEHI

>PotriWOX9b

PKPRWNPKPDQIRILEAIFNSGMVNPPRDEIRKIRVQLQEYGQVGDANVFYWFQNRKSRSKHRLRNLQNSKQHSSQQQKITSPTTKPVTANLAAPSSSSSSSEKSSPKGSKRTLSLSSPTFIDASNSPTSSVNKTYFQAHNEFVPEPFFFHSQQTGGGGTGAFAQGFCFSELSNMVHVQDHTVGPCSRLLLSEIMNSSASKKVNHEERNLKMQPQLCYTPVSPVTGSIGLAPPLTPSTDTSTFAFQTTINQIQGLGQSSGTTMLTVFINDVAFEVTMGPFNVREAFGDDVLLIQSSGQPVLTNECGVTLQSLQHGAFYYLVPFSMSEHILKKNGK

>AtWOX9

PKPRWNPKPEQIRILEAIFNSGMVNPPREEIRRIRAQLQEYGQVGDANVFYWFQNRKSRSKHKLRLLHNHSKHSLPQTQPQPQPQPSASSSSSSSSSSSKSTKPRKSKNKNNTNLSLGGSQMMGMFPPEPAFLFPVSTVGGFEGITVSSQLGFLSGDMIEQQKPAPTCTGLLLSEIMNGSVSYGTHHQQHLSEKEVEEMRMKMLQQPQTQICYATTNHQIASYNNNNNNNNIMLHIPPTTSTATTITTSHSLATVPSTSDQLQVQADARIRVFINEMELEVSSGPFNVRDAFGEEVVLINSAGQPIVTDEYGVALHPLQHGASYYLI

>AtWOX8

PKPRWNPKPEQIRILESIFNSGTINPPREEIQRIRIRLQEYGQIGDANVFYWFQNRKSRAKHKLRVHHKSPKMSKKDKTVIPSTDADHCFGFVNQETGLYPVQNNELVVTEPAGFLFPVHNDPSAAQSAFGFGDFVVPVVTEEGMAFSTVNNGVNLETNENFDKIPAINLYGGDGNGGGNCFPPLTVPLTINQSQEKRDVGLSGGEDVGDNVYPVRMTVFINEMPIEVVSGLFNVKAAFGNDAVLINSFGQPILTDEFGVTYQPLQNGAIYYLI

>PheWOX9

PKPRWNPRPEQIKILEGIFNSGMVNPPRDEIHRIRLQLQEYGQVGDANVFYWFQNRKSRTKNKLRAAGQLQPAGRAALARACASPSTPTSPAPVTPPRHLLAAPRSSGSSKSVKLTALTYAPAAAQGMLPATAIDLFSPTPSPALSACQLYYHSHPMPTPMVPVREFTSPEPLLLQWPQSQYLPATELGGVLGSHAHMPAMHPAVSTGVLLGLCNEELGQEAIDMSCSSKAIGQYLNTTCSTEPNIKTDAVSTVIREDEKARLGFLHYGFGVTTAAAAAVATSAPPAAPVHAAVASADDVSTAVLPCAAPSNVATGAVLTDQLQGLLDDGLIGGTAPPTATVVALGQDAVMCTSTAQYSVPAMMHLDVKLFGEAAVLLRHTGEPVLVDESGVTVEPLQQGAVYYVLVYICSL

>PheWOX9-like

MVNPPRDEIRRIRLQLQEYGQVGDANVFYWFQNRKSRNKNKLRAAGQLQPTGRAALARACAPPSTPTAPAPVTPPRHLLAAPVAPTSSSSSDRSSGSSKSVKLAALTSATAAAQGMLPATAIDLFSPAPSPALSACQLYYHSHPMAPTPTAPVRELTYPEPLLLQWPQSQYLPATELGGVLGSHAHTPAMNPAVSPSVLLGLCNEEPGQEIINMTCSSMALGQYRNTTCGTELSNKTDAVATMIREDEKARLGLLQCGFGVTAATSVPLAAPAHAAADDVSTAVLPSAAPSNAVTSAVLTDQLQGLLDVGLIGGTAPPTATVVAFARDAVMCTSTAQYSFPAMMHLDVKLFGEAAVLLRHTGEPVLVDESGVTVEPLQQGAVYYVLVSSLSRNIAVSCGLA

>OsWOX9

PKPRWNPRPEQIRILEGIFNSGMVNPPRDEIRRIRLQLQEYGQVGDANVFYWFQNRKSRTKNKLRAAGHHHHHGRAAALPRASAPPSTNIVLPSAAAAAPLTPPRRHLLAATSSSSSSSDRSSGSSKSVKPAAAALLTSAAIDLFSPAPAPTTQLPACQLYYHSHPTPLARDDQLITSPESSSLLLQWPASQYMPATELGGVLGSSSHTQTPAAITTHPSTISPSVLLGLCNEALGQHQQETMDDMMITCSNPSKVFDHHSMDDMSCTDAVSAVNRDDEKARLGLLHYGIGVTAAANPAPHHHHHHHHLASPVHDAVSAADASTAAMILPFTTTAAATPSNVVATSSALADQLQGLLDAGLLQGGAAPPPPSATVVAVSRDDETMCTKTTSYSFPATMHLNVKMFGEAAVLVRYSGEPVLVDDSGVTVEPLQQGATYYVLVSEEAVH

>GRMZMWOX9

PRPRWNPRPEQIRILEGIFNSGMVNPPRDEIRRIRLQLQEYGPVGDANVFYWFQNRKSRTKHKLRAAGQLQPSGSGRSALQARACAPAPVTPPRNLQLAAAAPVAPPTSSSSSSSDRSSGSSSSKSVTVTPTTAVALASPAGAAPAAVFRQQGVMPTTAMDLLTPLPSSSAALAARQLYYQYHSQIMAPAAPPMPDTVIASPEQFLPQWQQGGQQHYYLPATELGGVLDGHSHHTHEPPAAIHRPVSLSPSVLFGLCNEALRQDYCADISVVPTKGLGHGHQFWNSTTCGSDMGNSNSKIDAVSAVIRDDEKSRLGLLHYYGLAGATTTAAAAVAPAPLAADAAAGTATLLPSSAASDQLQGLLDAAGLLMGETPPTPTATVVAVARDAVTCAATATAQFSVPASMRLDVRLAFGEAALLARHTGEAVPVDESGVTVEPLQQDTLYYVLMQATNN

>BradiWOX8

TKARWSPRPEQIRILEAIFNAGVVNPPRDEIRRIRARLQEFGPVADANVFYWFQNRKSRTKHKLRQAAASAARASSSSSSPAPAPPVTPPPPRKHLVLGSNNSSSSSSSDRSSGSSSGGGKAASVVKPPSAAMAADLFAPLSGCSQLYYNRHPMLTAAPEPQPAPLTLQWPPPSQSQQQYSYYLPATELGGVVLGSGSGHAHAHAQAPSMASPGAAALLDGALGLTQDSIDGMSSYAAVKGPCDTNGQFSINAAAASVGVSDAMMSAAVFTEEEDKASWLGGGRLLHYASAAATSDVVSTAAPLSVNAAPSSALTDQLVLQELLDAGMIGGGGVPMATVVVMAAAGPDAGAAVQCYSVPAMARLDVARLFGEAALLLRHTGEPVPVDAARGVTLDHGALYYVLV

>OsWOX8a

PKPRWNPRPEQIRILEAIFNSGMVNPPRDEIPRIRMQLQEYGQVGDANVFYWFQNRKSRSKNKLRSGGTGRAGLGLGGNRASAPAAAHREAVAPSFTPPPPILPAPQPVQPQQQLVSPVAAPTSSSSSSSDRSSGSSKPARATSTQAMSVTTAMDLLSPLAAACHQQMLYQGQPLESPPAPAPKVHGIVPHDEPVFLQWPQSPCLSAVDLGAAILGGQYMHLPVPAPQPPSSPGAAGMFWGLCNDVQAPNNTGHKSCAWSAGLGQHWCGSADQLGLGKSSAASIATVSRPEEAHDVDATKHGLLQYGFGITTPQVHVDVTSSAAGVLPPVPSSPSPPNAAVTVASVAATASLTDFAASAISAGAVANNQFQGLADFGLVAGACSGAGAAAAAAAPEAGSSVAAVVCVSVAGAAPPLFYPAAHFNVRHYGDEAELLRYRGGSRTEPVPVDESGVTVEPLQQGAVYIVVM

>OsWOX8b

PKPRRNPRPEQIRILEAIFNSGMVNPPRDEIPRIRMQLQEYGQVGDANVFYWFQNRKSRSKNKLRSGGTGRAGLGLGGNRASEPPAAATAHREAVAPSFTPPPILPPQPVQPQQQLVSPVAAPTSLSSSSSDRSSGSSKPARATLTQAMSVTAAMDLLSPLRRSARPRQEQRHV

>PheWOX8

PKPRWNPRPEQIRILEAIFNSGMVNPPRDEIPRIRMQLQEYGQVGDANVFYWFQNRKSRSKNKLRAAAGRAAVARACAPAREAAAPFTPPPPSPQQVQPQQLLVSPVAPTSSSSSSDRSSGSSKSVKPAAQAMSVTAAMDLLSPLAAACHQQMHYQGQPVAPAPAPAPNKVQELVATDEAIFLQWPQGSGHCLSAAELAAILGAQYMNVPVQQPPAASPAGMFLGLCNEVAAGSNITGHRNSAGLGQYWTSGADQLVLGKSSAASNTAVAREEAHEDATKLGLLQYGFGVSAPALDAHATSAAAALLPVASAPDAVVTVASLAASTIANGAVANNQLQAGLAAAVFTGAAAAAAAAPTGTVAVARGPAVVCIAGTSAVYSFPATHLDVTRMFGEAAVLFRYNGEAVLVDEFGVTVEPLQQGAVYCVLI

>PheWOX8-like

MVNPPRDEIPRIRMQLQEYGQVGDANVFYWFQNRKSRSKNKLRAAAGRAPLTRACAPAREAAAPFTPSPPPPKQVQPQQLLVSPVAPTSSSSSSSDRSSGSSKPVEPAAQPMSAAAAMDQLSPLAAACHQQMHYHGHPVAPAPVAAPKVQELEATDEPIFLQKPQGHCLSAAELAAILGEQYMHVPVQQPPPASPTGMFLGLYNEVAAGPTTTGHRSCAWDAGLGQYWPSGADQLVLGKSSAASNTAVARQEAHEGATKLGLLQYGFGVSAPTLDATSPAAALLPAASPDDAVTVASLAAGTIANGTGAKNQFQGLADVGYTGAATATTATVAVARGDAVVCIAGTSAFYTVPATHLDVVKMFGEAAVLFRYNGEPVLVDEFGLTVEPLQQGAVYCVLI

>BradiWOX9

PKPRWNPRPEQIRILEALFNSGMANPPRDEIPRIRMKLQEYGPVGDANVFYWFQNRKSRSKNKLLRAAGSGAASRAGAAPARACAPAARQHAAASPYTTPQPKQQQLQAPHVSPTMMAPTSSSSSSSDRSSGSSKPVKPTTAAMDLLSPLAAACHQQMHYQPLGLGLQPATVSAPAATAALEEFVPTATDVEPIFLQYPQGHCLSAGELAAILGAQYMHAPVQQPAPAPASPAGMLLGLCNELAAGPTRSGAWIGAGGLGQHWPSGADQLGLGKSSETFNASAVATDVAHEDATKLGLLHYGFGLSSPPAVNAATTSAPTAVLPLPASSPETGAVTVASAAAAAAGLSNLFATTTAATSEAVTYSHLQEGEAEAADVGFAGTGVVPRGAAVVCIAGTNAVCNVPAGHLHVKTYFGEGAVLARFRSGRFEPLAVDASLGLTVEPLQHGDIYYCVLI

>GRMZMWOX8b

PKPRWNPRPEQIRILEAIFNSGMVNPPRDEIPRIRMRLQQYGQVGDANVFYWFQNRKSRSKNKLRSSTAGTGRLGLQGLARAPGRGAAAAPPPVEPPPLVQNQFHMLASPAQAPTSSSSSSSDRSSGSSKPAAEPAMPATAAPMDLLGPLAAACPQMYYQGSPVAPAHKVLDLVASVEPVFQPWPQGYCLSAAEVATILGGQYMHVPVQQQPPAPLPAGALLGLCNDVTEPTAVVTGHKTCAWGPAGLGQSWPCGGADHHQPGKNNNTAARELAHEDDATKLGLLQYGFGATTAMEAAPAVAPLAASPAGGAVTMASVSASTAGLTGFPASTNGVVANYDLLQGLAVPGGGAGAGRAPAAVAVAADAAPTAAQEGVVALCITDSITGKSVAHNVAAARLDVRAQFGEAAVLLRCGGERGLDLEPVPVDASGCTVEPLQRGAFYYVLL

>GRMZMWOX8c

PKPRWNPRPEQIRILEAIFNSGMVNPPRDEIPRIRMRLQEYGQVGDANVFYWFQNRKSRSKNKQRTGQLGLGLARAPGCGAAAPPVTPQPLIQNQFQVLASPAQAPASSSSSSSDRSSGSSKPAPQPMSATAAAMNFPGPLGAACAQMYYQAHPVAPVSALPAHKVQDPVASDEPVFQPWPQGYFLSAAEVASILGGQYRHDVPVQQQPPATLPAGAFLGLYNEVTEPTVTGHRTCAWGPAGLGQFWPVGGADHHQHHKHNTTAATNTVARDAAHEHATTLGLLQYGFEASAAMETASAAVPLAASPGTAASVATAGLTSLPASTNAVVVNYDLLQGLAVPGSGSGAVGVSTGGAPPVAVAAAPTAAQEGVVVALCITDSVTGKSVAHNVAAARLDVRAQFGEAAVLLRAVGDRGGLDLVPVPVDALGCTVEPLQHGAFYYVLV

>GRMZMWOX8a

PKPRWNPRPEQIRILEAIFNSGMVNPPREEIPRIRMRLQEYDQQQPPAALPAGAFLGLCNEVTEPTVTCHRTCAWGPAGLGQFWPVGGADHHQHHKNNTTTVTNTVASDAAHEHNTTLGLLQYGFGASVAMETTSAAVSLAASRGTATSMATTRLTSFPTSTNVVVVNYDLLQGSFILSVTWRGSQRIFYSHTVLTQKSPLGTVWIAAHFELFEQCQILSSIWWRLKSAVLSMASGANAVESCDTSSIIMQQVGWQCLRADYTELRSRAMKLAELLRQAARVELYERPAARIMADMERALHKAAGMATRCFQSHSRRSHTPFIYPYLLGFSISCFLLRMDYGLWTKNCCSTYNKWDGVLPSKI
